# Massively parallel unsupervised single-particle cryo-EM data clustering via statistical manifold learning

**DOI:** 10.1371/journal.pone.0182130

**Published:** 2017-08-07

**Authors:** Jiayi Wu, Yong-Bei Ma, Charles Congdon, Bevin Brett, Shuobing Chen, Yaofang Xu, Qi Ouyang, Youdong Mao

**Affiliations:** 1 State Key Laboratory for Artificial Microstructure and Mesoscopic Physics, Institute of Condensed Matter Physics, School of Physics, Center for Quantitative Biology, Peking University, Beijing, China; 2 Intel Parallel Computing Center for Structural Biology, Dana-Farber Cancer Institute, Boston, Massachusetts, United States of America; 3 Software and Services Group, Intel Corporation, Santa Clara, California, United States of America; 4 Department of Biophysics, Peking University Health Science Center, Beijing, China; 5 Peking-Tsinghua Joint Center for Life Sciences, Peking University, Beijing, China; 6 Department of Microbiology and Immunobiology, Harvard Medical School, Boston, Massachusetts, United States of America; Soochow University, CHINA

## Abstract

Structural heterogeneity in single-particle cryo-electron microscopy (cryo-EM) data represents a major challenge for high-resolution structure determination. Unsupervised classification may serve as the first step in the assessment of structural heterogeneity. However, traditional algorithms for unsupervised classification, such as K-means clustering and maximum likelihood optimization, may classify images into wrong classes with decreasing signal-to-noise-ratio (SNR) in the image data, yet demand increased computational costs. Overcoming these limitations requires further development of clustering algorithms for high-performance cryo-EM data processing. Here we introduce an unsupervised single-particle clustering algorithm derived from a statistical manifold learning framework called generative topographic mapping (GTM). We show that unsupervised GTM clustering improves classification accuracy by about 40% in the absence of input references for data with lower SNRs. Applications to several experimental datasets suggest that our algorithm can detect subtle structural differences among classes via a hierarchical clustering strategy. After code optimization over a high-performance computing (HPC) environment, our software implementation was able to generate thousands of reference-free class averages within hours in a massively parallel fashion, which allows a significant improvement on *ab initio* 3D reconstruction and assists in the computational purification of homogeneous datasets for high-resolution visualization.

## Introduction

Single-particle cryo-EM is evolving into a mainstream approach in visualizing the three-dimensional (3D) structures of biomolecules in their native functional states at near-atomic resolutions [1,2]. Since individual biomolecules may sample multiple conformations, a prerequisite for high-resolution structure determination is to obtain a ‘pure’ dataset that only includes particle images in the same conformational state [3]. To distinguish different conformations in raw particle images, unsupervised two-dimensional (2D) clustering is commonly used as the first step in the evaluation of structural heterogeneity [1,4–6], or used as an intermediate step during the alteration of 2D and 3D classifications for *in silico* purification [7,8]. Besides, high-quality unsupervised 2D class averages are essential for reliable initial reconstruction and its verification [1,9,10].

Although several classification schemes were proposed for single-particle analysis [11–15], there are mainly two approaches for unsupervised 2D clustering of single-particles implemented in multiple end-user software packages [16–21] and widely used in cryo-EM structure determination: (1) K-means clustering following reference-free alignment through cross-correlation (CC) and multivariate statistical analysis (MSA) [1,22–24], and (2) unsupervised maximum-likelihood (ML) or *maximum a posteriori* (MAP) classification [25–27]. In the former approach, the classification accuracy is affected by the noise-induced misalignment resulting from false peaks in cross-correlation computation. Noise may also introduce errors in the distance calculation in K-means clustering. Hence, its performance is dramatically reduced as the signal-to-noise ratio (SNR) decreases. By contrast, the ML-based approach explores optimal probability in measuring image similarity, and exhibits robust resistance to noise-induced misalignment [27]. However, a prominent drawback lies in that the likelihood matching insufficiently differentiates structural heterogeneity among similar but critically different views. In each ML-classified group of single-particles one could find a mixture of heterogeneous 2D projection structures with a large variation of likelihood. This effect causes a decrease in the effective number of classes with increasing the number of iterations during ML optimization. To date, there has been a lack of unsupervised single-particle clustering methods that can efficiently sort out highly heterogeneous single-particles into thousands of homogenous 2D classes while still keeping computation efficient [5].

Manifold learning encompasses the disciplines of geometry, computation, and statistics, and has become an important research frontier in data mining and machine learning. It is a class of algorithms devised for recovering a low-dimensional manifold embedded in a high-dimensional data space. Perhaps the most popular algorithm for linear dimensionality reduction is principal component analysis (PCA). Given a data set, PCA finds the directions along which the data have maximum variance in addition to the relative importance of these directions. Most real-world high-dimensional data are intrinsically governed by hidden variables through nonlinear relationships. When linear approximation in dimensionality reduction fails in finding a good low-dimensional representation of high-dimensional data, nonlinear dimensionality reduction can be exploited as an alternative solution. Major breakthroughs in methods for recovering low-dimensional nonlinear embeddings of high-dimensional data [28,29] have led to the construction of a number of algorithms carrying out nonlinear manifold learning. Unlike PCA, nonlinear manifold learning attempts to use nonlinear kernel functions or mapping to find the directions along which the data have significant variance. To date, several nonlinear manifold learning frameworks have been proposed, such as isometric feature mapping (Isomap) [28,30–32], generative topographic mapping (GTM) [33–35], locally linear embedding (LLE) [29,36,37], and semidefinite embedding (SDE) [38]. Furthermore, several algorithms for manifold learning have been successfully applied to high-dimensional data for specific tasks, such as discriminant image clustering [39], image feature extraction [40] and person-independent precise 3D pose estimation [41].

Recently, manifold embedding via diffusion map was applied to study the continuous conformational changes of the 80S ribosome by single-particle cryo-EM [42]. In this approach, a one-dimensional manifold was used to describe a continuous conformational spectrum that can be discerned through a narrow angular aperture. The approach begins with a classification of data into different orientations with respect to a common 3D reference, with the assumption that the changes in the structure are relatively small so that a projection-matching based refinement algorithm with a single 3D reference can be used. However, this assumption does not necessarily hold for molecules exhibiting dramatic conformational changes, in which a small angular aperture is not sufficient to distinguish the conformational spectrum. Hence, such an approach is yet to be adapted for more general conditions.

Here we introduce an unsupervised clustering method for single-particle cryo-EM data based on GTM [33–35], which is a framework for statistical manifold learning, without any assumptions regarding how the molecules assume their conformations in the cryo-EM data [33–35]. We implemented the GTM-based unsupervised single-particle clustering algorithm in a software package named ROME (Refinement and Optimization based on Machine lEarning), which has been optimized for both Intel^®^ Xeon^®^ multi-core processors and Intel^®^ Many-Integrated Core (MIC) architecture [43] to enable efficient computation of thousands of reference-free class averages in a highly affordable fashion. Algorithmic implementations of unsupervised clustering methods in the existing software packages [16–21] normally allow the efficient computation of tens to a few hundred of classes. In this study, through adapting GTM to cryo-EM image data and optimizing its computational efficiency against Intel^®^ MIC architecture, we can achieve unsupervised data clustering with the number of classes on the order of magnitude of 10^3^ or higher within several hours in a highly parallel fashion. Our unsupervised clustering approach can markedly improve the quality and resolution of *ab initio* 3D models with angular reconstitution[44]. We further tested our approach using several cryo-EM datasets to demonstrate the advantage of GTM-based unsupervised clustering in discerning subtle structural differences directly from 2D class averages corresponding to distinct conformations.

## Methods

### Mathematical model for statistical manifold learning

**Fig 1:**
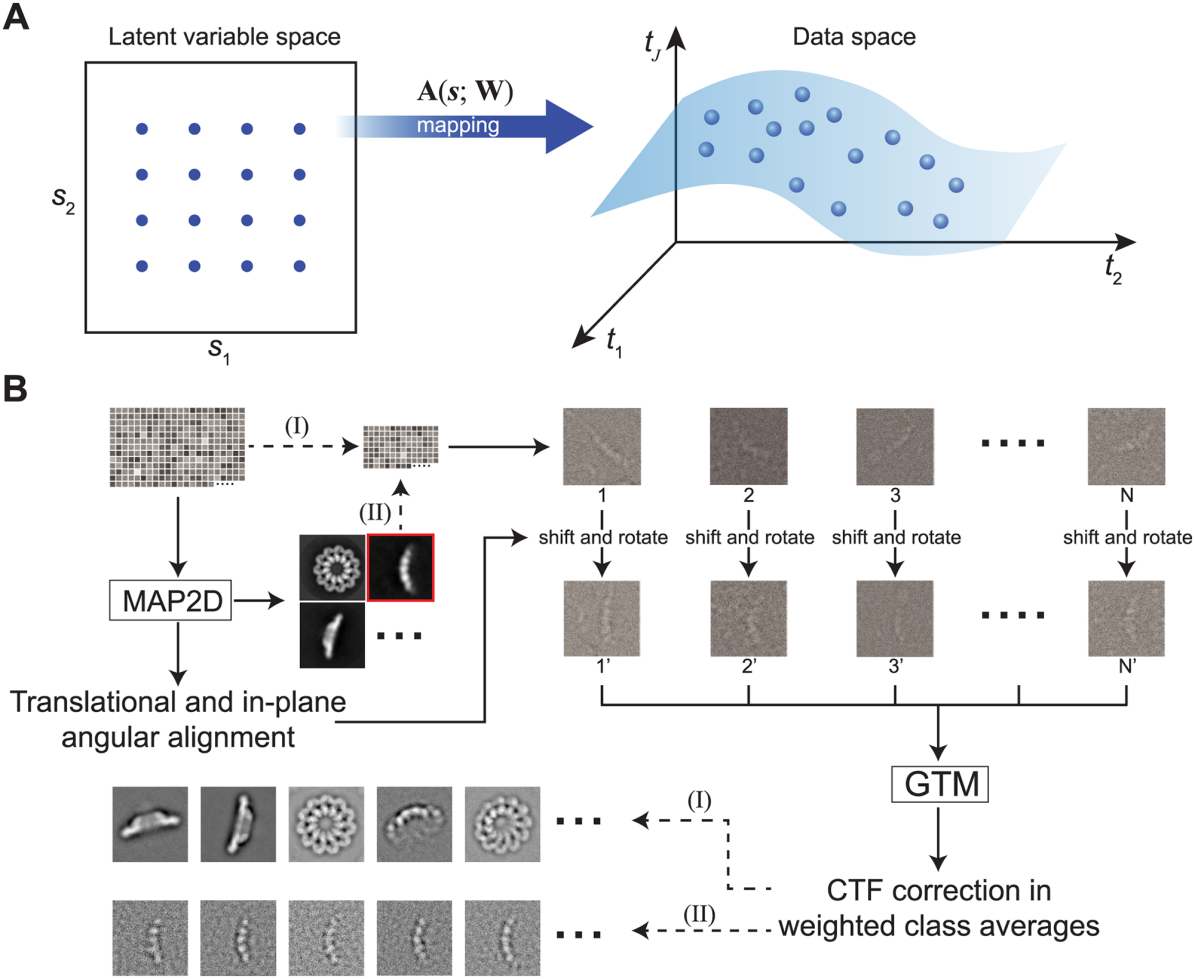
Strategy for unsupervised single-particle clustering via statistical manifold learning. **(A)** The fundamental principle of GTM is to establish a numerical relationship between variables in the latent space and a non-Euclidean manifold composed of the Fourier transformed image data in the data space. The manifold embedding can be determined by a set of nonlinear basis functions and a weighted parametric matrix. The likelihood function for the nonlinear mapping is solved by the expectation-maximization algorithm. **(B)** The workflow of implementing the unsupervised clustering strategies in ROME is as follows: (I) All images are aligned using MAP2D in a reference-free manner, and are subsequently classified into many groups by unsupervised GTM. (II) The unsupervised classes obtained in step (I) are further classified into many sub-classes by unsupervised GTM in a hierarchical fashion.

The goal of GTM is to find a representation for the distribution *p*(***t***) of a dataset in a *J*-dimensional data space ***t*** = (*t*_1_, …, *t*_*J*_) in terms of *L*-dimensional latent variables ***s*** = (*s*_1_, …, *s*_*L*_). One may consider a non-linear, parametric function ***t =* A**(***s***; **W**), which maps points ***s*** in the latent space into corresponding points **A**(***s***; **W**) in the data space [33] (Fig 1A). The mapping is controlled by a set of parameters **W**, which represent the weights and biases in the case of a feed-forward neural network as the mapping. In the situation in which the dimensionality *L* of the latent space is less than the dimensionality *J* of the data space, the nonlinear transformation **A**(***s***; **W**) maps the latent space onto an *L*-dimensional non-Euclidean manifold S embedded within the data space. Previous studies have established GTM as an alternative to self-organizing maps (SOM) [45] and that the GTM framework overcomes most limitations of SOM while introducing no significant disadvantages [33,34].

To adapt the general framework of GTM to the single-particle cryo-EM data clustering problem, we built the contrast transfer function (CTF) into the non-linear mapping function in Fourier or reciprocal space, which allows for an efficient correction of the aberration effect of the objective lenses in electron microscopy [1]. A vector in data space ***x***_*i*_ represents the Fourier transform of a particle image. A latent variables ***s*** reflects the inherent degrees of freedom controlling the 2D structural differences observed in single-particle images, which arise either from distinct conformational states of the imaged biomolecules or from different viewing angles. For the sake of simplicity, we used ***t***_*i*_ to represent the translated and rotated version of ***x***_*i*_, so that ***t***_*i*_ = *T*_*-τ*_(***x***_*i*_), where *T*_*-τ*_ denotes the operator of in-plane image rotation and translation; *τ* = (*θ*, *r*_*x*_, *r*_*y*_) is the in-plane rotation angle and translation, which can be determined through a 2D image alignment procedure, such as the regularized ML method [25–27]. Thus, an image ***t***_***i***_ in the frequency domain can be modelled as:

$$t_{ij}={\text{CTF}}_{ij}A_{j}\left(s_{k};W\right)+n_{ij}$$ (1)

where *t*_*ij*_ is the *j*-th component of the 2D Fourier transform of the *i*-th experimental image; CTF_*ij*_ is the *j*-th component of the contrast transfer function for the *i*-th image; *n*_*ij*_ is Gaussian noise; and *A*_*j*_(***s***_*k*_; **W**) is the *j*-th component of the 2D Fourier transform of the underlying *k*-th molecular projection.

The nonlinear function **A**(***s***; **W**) is expanded by a set of basis functions {*ϕ*_1_, *ϕ*_2_, …, *ϕ*_*M*_} through the weight matrix **W**:

A(s;W)=Φ(s)W (2)

where **A**(***s***; **W**) is a *K × J* matrix with elements *A*_*j*_(***s***_*k*_; **W**); **Ф**(***s***) is a *K × M* matrix with elements *ϕ*_*km*_ = *ϕ*_*m*_(***s***_*k*_); and **W** is an *M × J* matrix containing the weight and bias parameters. In our algorithmic design, we used a combination of one fixed basis function and many Gaussians basis functions in the form:

$$\phi_{m}\left(s_{k}\right)=\{\begin{array}{c} \text{exp}\left\{-\frac{{\left\|s_{k}-\mu_{m}\right\|}^{2}}{2\sigma^{2}}\right\},m\leq M_{NL}\\ 1,m=M_{NL}+1 \end{array}$$ (3)

where *M*_*NL*_ is the number of Gaussian basis functions; *μ*_m_ is the mean of the Gaussian distribution; and *σ* is the variance of the Gaussian distribution.

Although the noise in cryo-EM data may take multiple forms, potentially including both white and non-white noises, previous studies have established that cryo-EM noise can be largely approximated with the normal distribution without obvious detrimental effects on data analysis [1,25–27]. Thus, in our algorithm we chose the Gaussian distribution for the probability density function in the data space:

$$p\left(t_{i}|s,W,\beta \right)=\overset{J}{\underset{j=1}{\prod }}{\left(\frac{\beta_{ij}}{2}\right)}^{\frac{1}{2}}\text{exp}\left\{-\frac{\beta_{ij}}{2}{\left(t_{ij}-{\text{CTF}}_{ij}A_{j}\left(s;W\right)\right)}^{2}\right\}$$ (4)

where 1/*β*_*ij*_ is the variance of noise for the *j*-th pixel of the *i*-th image.

Since we manage to classify a dataset consisting of *N* images {***t***_1_, ***t***_2_, …, ***t***_*N*_} into *K* classes, this can be translated into the problem of finding *K* points {***s***_1_, ***s***_2_, …, ***s***_*K*_} in the latent space that are mapped onto {***t***_1_, ***t***_2_, …, ***t***_*N*_} in the data space through Eq (1). To make the problem tractable, we considered a specific form for the probability distribution *p*(***s***) given by a sum of delta functions centered on *K* nodes of a regular grid in the latent space:

$$p\left(s\right)=\frac{1}{K}\overset{K}{\underset{k=1}{\sum }}\delta \left(s-s_{k}\right)$$ (5)

By integrating over the ***s***-distribution on the manifold, the distribution *p*(***t***_*i*_|**W**, **β**) in the data space for a given value of **W** is:

$$p\left(t_{i}|W,\beta \right)=\int p\left(t_{i}|s,W,\beta \right)p\left(s\right)ds=\frac{1}{K}\overset{K}{\underset{k=1}{\sum }}p\left(t_{i}|s_{k},W,\beta \right)$$ (6)

The joint probability density of observing the collection of *N* images **t** = {***t***_1_, ***t***_2_, …, ***t***_*N*_} is $p\left(t|W,\beta \right)=\prod_{i=1}^{N}p\left(t_{i}|W,\beta \right)$. The ML estimate of the model parameters **Θ** = {**W**, **β**} can be found by maximizing the logarithmic form of the joint probability, namely, $\hat{\Theta }=\text{arg }{\text{max}}_{\Theta }\sum_{i=1}^{N}\text{ln}p\left(t_{i}|\Theta \right)$. As we would like to consider the prior probability of the weight matrix, a regularized ML estimator, also called the *maximum a posteriori* (MAP) estimate, with respect to **Θ** can be used to describe the nonlinear mapping problem. Thus, the model parameters are sought to maximize the MAP:

$$\hat{\Theta }=\text{arg}\underset{\Theta }{\text{ max}}\left[\overset{N}{\underset{i=1}{\sum }}\text{ln}p\left(t_{i}|\Theta \right)+\text{ln}p\left(\Theta \right)\right]$$ (7)

Here we assume a Gaussian prior distribution over **W**:

$$p\left(W\right)={\left(\frac{\alpha }{2\pi }\right)}^{W/2}\text{exp}\left(-\frac{\alpha }{2}{\left\|W\right\|}^{2}\right)$$ (8)

Where 1/*α* is the variance of the Gaussian prior distribution over the weight matrix **W**, and *W* is the total number of elements in the matrix **W**.

### Expectation-maximization algorithm

The unsupervised clustering problem is ill-posed due to a high level of noise, potentially missing orientations and discontinuity between different conformational states in single-particle cryo-EM data. To enable effective computation, the expectation-maximization (E-M) algorithm can be used to estimate the model parameters **Θ** corresponding to the optimal clustering. The E-M algorithm alternates between two steps: the expectation step (E-step) and the maximization step (M-step). In the E-step, the old model parameters **W**^(*n*)^ and are used to evaluate the posterior probabilities, or responsibilities, *R*_*ki*_(**Θ**^(*n*)^) of each latent variable ***s***_*k*_ for every image ***t***_*i*_ using Bayes’ theorem:

$$R_{ki}\left(\Theta^{\left(n\right)}\right)=p\left(s_{k}|t_{i},W^{\left(n\right)},\beta^{\left(n\right)}\right)=\frac{p\left(t_{i}|s_{k},W^{\left(n\right)},\beta^{\left(n\right)}\right)p\left(s_{k}\right)}{\sum_{k^{\prime }=1}^{K}p\left(t_{i}|s_{k\prime },W^{\left(n\right)},\beta^{\left(n\right)}\right)p\left(s_{k\prime }\right)}$$ (9)

In the M-step, *Q*(**Θ, Θ**^(*n*)^) is maximized with respect to the model parameters **Θ**:

$$Q\left(\Theta ,\Theta^{\left(n\right)}\right)=\overset{N}{\underset{i=1}{\sum }}\overset{K}{\underset{k=1}{\sum }}R_{ki}\left(\Theta^{\left(n\right)}\right)\text{ln}p\left(t_{i}|\Theta \right)+\text{ln}p\left(\Theta \right)$$ (10)

To this end, the partial derivatives of Eq (10) with respect to each model parameter should equal zero when *Q*(**Θ, Θ**^(*n*)^) is maximized. This allows us to estimate the model parameters iteratively. Maximizing *Q*(**Θ, Θ**^(*n*)^) with respect to **W** thus gives rise to the following equation, which uses $R_{ki}\left(\Theta^{\left(n\right)}\right),\;\beta_{ij}^{\left(n\right)}$ to update the weight matrix **W**^(*n*+1)^ in the (*n*+1)-th iteration:

$$\overset{N}{\underset{i=1}{\sum }}\overset{K}{\underset{k=1}{\sum }}R_{ki}\left(\Theta^{\left(n\right)}\right)\beta_{ij}^{\left(n\right)}\phi_{m}\left(s_{k}\right){\text{CTF}}_{ij}\left(t_{ij}-{\text{CTF}}_{ij}\overset{M}{\underset{m^{\prime }=1}{\sum }}\phi_{m^{\prime }}\left(s_{k}\right)W_{m^{\prime }j}^{\left(n+1\right)}\right)-\alpha W_{mj}^{\left(n+1\right)}=0$$ (11)

Similarly, by maximizing Eq (10) with respect to **β**, we obtained the following re-estimation formula that uses *R*_*ki*_(**Θ**^(*n*)^) and **W**^(*n*+1)^ to update $\beta_{ij}^{\left(n+1\right)}$:

$$\frac{1}{\beta_{ij}^{\left(n+1\right)}}=\overset{K}{\underset{k=1}{\sum }}R_{ki}\left(\Theta^{\left(n\right)}\right){\left(t_{ij}-{\text{CTF}}_{ij}A_{j}\left(s_{k};{\text{W}}^{\left(n+1\right)}\right)\right)}^{2}$$ (12)

During the iteration of the E-M algorithm, no class averages need to be computed. Nonetheless, upon convergence of the E-M algorithm, one can calculate the class averages using the posterior probability *R*_*ki*_:

$$A_{kj}=\frac{\sum_{i=1}^{N}R_{ki}\left(\Theta \right)\beta_{ij}{\text{CTF}}_{ij}t_{ij}}{\sum_{i=1}^{N}R_{ki}\left(\Theta \right)\beta_{ij}{\text{CTF}}_{ij}^{2}+\alpha }$$ (13)

Note that this equation is derived from Eqs (11) and (12) and resembles the Wiener filter.

Note that Eq (11) is a system of linear equations. Solving this system of equations is equivalent to separately solving *J* systems of linear equations **C**_*j*_***w***_*j*_ = ***b***_*j*_, *j* = 1, …, *J*, where

$$C_{j}=\left(\begin{array}{ccc} \sum_{i=1}^{N}\sum_{k=1}^{K}R_{ki}\left(\Theta^{\left(n\right)}\right)\beta_{ij}^{\left(n\right)}\phi_{1}^{2}\left(s_{k}\right){\text{CTF}}_{ij}^{2}+\alpha & \cdots & \sum_{i=1}^{N}\sum_{k=1}^{K}R_{ki}\left(\Theta^{\left(n\right)}\right)\beta_{ij}^{\left(n\right)}\phi_{1}\left(s_{k}\right){\text{CTF}}_{ij}^{2}\phi_{m}\left(s_{k}\right)\\ \vdots & \ddots & \\ \sum_{i=1}^{N}\sum_{k=1}^{K}R_{ki}\left(\Theta^{\left(n\right)}\right)\beta_{ij}^{\left(n\right)}\phi_{m}\left(s_{k}\right){\text{CTF}}_{ij}^{2}\phi_{1}\left(s_{k}\right) & \cdots & \sum_{i=1}^{N}\sum_{k=1}^{K}R_{ki}\left(\Theta^{\left(n\right)}\right)\beta_{ij}^{\left(n\right)}\phi_{m}^{2}\left(s_{k}\right){\text{CTF}}_{ij}^{2}+\alpha \end{array}\right)$$

$w_{j}=\left(\begin{array}{c} W_{1j}\\ \vdots \\ W_{Mj} \end{array}\right)$, $b_{j}=\left(\begin{array}{c} B_{1j}\\ \vdots \\ B_{Mj} \end{array}\right)$, and $B_{mj}=\sum_{i=1}^{N}\sum_{K=1}^{K}R_{ki}\left(\Theta^{\left(n\right)}\right)\beta_{ij}^{\left(n\right)}\phi_{m}\left(s_{k}\right){\text{CTF}}_{ij}t_{ij}$. This decomposition allows us to distribute the numerical computation of Eq (11) to many computer nodes in parallel. To further reduce the computational cost, we used a mean to replace $\beta_{ij}^{\left(n+1\right)}$ in the numerical solution of Eq (11), as follows:

$$\bar{\beta_{ij}^{\left(n+1\right)}}=\frac{N\times J}{\sum_{i=1}^{N}\sum_{j=1}^{J}\frac{1}{\beta_{ij}^{\left(n+1\right)}}}$$ (14)

where *N* is the total number of the images and *J* is the total number of pixels in each image.

The convergence of the E-M algorithm can be monitored by the weighted loss function

$$L^{\left(n\right)}=\overset{N}{\underset{i=1}{\sum }}\overset{J}{\underset{j=1}{\sum }}\overset{K}{\underset{k=1}{\sum }}R_{ki}\left(\Theta^{\left(n\right)}\right){\left(t_{ij}-{\text{CTF}}_{ij}A_{j}\left(s_{k};W^{\left(n\right)}\right)\right)}^{2}$$ (15)

Thus, a convergence criterion can be devised based on the weighted loss function $L^{\left(n\right)}$, i.e., the program is terminated when $L^{\left(n\right)}$ does not change more than 0.1% for six consecutive iterations.

### Implementation of the GTM algorithm

Based on the above mathematical framework, we have implemented the following algorithm in the ROME software package.

**Input**: Particle images ***t***_*i*_, and their corresponding **CTF**_*i*_, *i* = 1, 2, 3, …, *N*.

**Output**: Class assignment and class averages ***A***_*k*_

1. Set the fixed number of latent points {***s***_*k*_}, *k* = 1, 2, 3… *K*. The space between two adjacent points is 1.
2. Set the values of basis function centers{*μ*_*m*_}, m = 1, 2, 3… *M*. $\mu_{m}=\frac{M}{M-1}+\frac{\left(K-1\right)mM}{{\left(M-1\right)}^{2}}$
3. Select the value of basis function width $\sigma =\frac{\left(K-1\right)M}{{\left(M-1\right)}^{2}}$.
4. Calculate the basis function *ϕ* from Eq (3).
5. Initialize the weighted matrix **W** from a Gaussian circle and **β**; and set *α* to a fixed value of 0.01.
6. Iterate until the convergence criterion, $\left|\frac{L^{\left(n-i\right)}-L^{\left(n-i-1\right)}}{L^{\left(n-i-1\right)}}\right|<0.1\%$ (*i* = 0, 1, …, 5), is satisfied:
  - Set iteration number *n* = *n* + 1;
  - E-step:
    - Compute the *δ*_*ij*_ distance between the latent space and data space
      $$\delta_{ij}={\left(t_{ij}-{\text{CTF}}_{ij}\sum_{m=1}^{M}\phi_{m}\left(s_{k}\right)W_{mj}\right)}^{2};$$
    - Compute the probability matrix **P** from Eq (4) using *ϕ*, *δ*_*ij*_ and **W**;
    - Compute the responsibility matrix **R** from Eq (9) using **P**, *δ*_*ij*_ and **W**.
  - M-step:
    - Use Eq (11) and **R** to update **W**;
    - Use **R** and *δ*_*ij*_ to update *β*_*ij*_ and calculate its mean from Eq (14).
7. Calculate class averages from Eq (13).

### Hierarchical strategy for unsupervised clustering

Although our GTM-based algorithm may be advantageous for unsupervised classification, it is not computationally efficient for image alignment, a procedure that determines three geometrical parameters for each image: *x*-*y* translation and in-plan rotation. Thus, we employed the adaptive MAP-based 2D alignment method (hereafter referred to as MAP2D) [19,46] to align single-particle images prior to the GTM-based clustering procedure (Fig 1B). Averages from random subsets of unaligned images were used to initialize the MAP2D-based image alignment. Furthermore, a Gaussian model was used to initialize the parameters **W** of the E-M algorithm in GTM solution. Thus, in both steps of the MAP2D-based alignment and GTM-based clustering, no external initial model or reference was needed, ensuring the unsupervised nature of our approach.

The unsupervised single-particle clustering may be conducted in a hierarchical fashion. First, all particles are aligned based on the translations and rotations determined by MAP2D. Then GTM is applied to partition these shifted and rotated particles into different classes. However, some classes might be mixtures of non-identical conformational states. For these classes, the aligned particles in each class can be further classified into tens or hundreds of reference-free sub-classes by GTM clustering. This hierarchical clustering strategy can be further iterated and used in different scenarios. For example, if large numbers of classes are needed to assess sample heterogeneity, the strategy can be used to obtain relatively more unsupervised classes without any human intervention. If one needs to verify the structural homogeneity within individual classes, the hierarchical clustering strategy can help examine structural variability within specific classes at a deeper level.

### Comparative benchmarking with simulated data

Each simulated dataset, including 50,000 projections, was generated by projecting the density map of the *Escherichia coli* 70S ribosome along random orientations [20]. We uniformly chose 500 orientations covering half a sphere. Each orientation was regarded as a Gaussian center, around which 100 projections were generated with a Gaussian distribution. To simulate realistic situations, we added random shifts in the range of -2.0 to 2.0 pixels relative to the image center. We further generated defocus values in the range of -1.0 to -3.5 μm and modified the projection images with the CTF in the Fourier domain. The projections were then additively contaminated with Gaussian noise at SNRs of 1/50, 1/100, 1/150, and 1/200, which allowed us to benchmark the proposed algorithm at different noise levels.

Using the simulated data, we compared the results from our approach with those obtained using RELION [19], EMAN2 [47] and SPIDER [20]. All procedures were set to classify these 50,000 particles into 500 classes. In all cases described below, the alignment or classification was run for 30 rounds. Both in our approach and in the unsupervised 2D classification in RELION, we set up an in-plane rotational search with an angular increment of 2.5°, a translational search in the range of -3 to 3 pixels, and 100 classes for MAP2D alignment. We also conducted a classification based on an alternative method in RELION, which was implemented using the ‘skip_align’ option to classify pre-aligned particles without doing further alignment during classification. To enable a fair comparison, in the pre-alignment step, we set up the same control parameters for MAP2D alignment as the ones without using the ‘skip_align’ option in RELION.

EMAN2 uses multivariate statistical analysis (MSA) [1,22–24] with multi-reference alignment (MRA) method [24,47]. PCA was used in the MSA to reduce the dimensionality of images [47]. The particles were first CTF-corrected through phase flipping. Then, translational and rotational invariants were calculated and used for initial clustering. Next, PCA in the MSA step was used to capture the principal features, which were then used as multiple references for image alignment and K-means clustering in the MRA step. We set up 20 MSA basis vectors, 12 alignment references and 8 iterations for the EMAN2 procedure.

SPIDER combines reference-free alignment (RFA) with PCA and K-means clustering [48]. It involves three steps. First, all CTF-corrected noisy images were filtered through a low-pass Butterworth filter, with the pass band and stop band frequencies at 0.08 and 0.12, respectively. These particle images were then aligned globally in a reference-free manner, in order to find rotations and translations for all images that minimize the sum of squared deviations from their mean using the AP SR command in SPIDER. Second, PCA was used to solve the eigenvalue and eigenvector of all rotated and shifted images in the MSA step. Third, all particles were classified by K-means clustering [20].

### Experimental cryo-EM datasets

We used three experimental cryo-EM datasets to examine if our clustering approach could yield useful classes and detect subtle structural difference among classes. The experimental cryo-EM datasets were collected using an FEI Tecnai Arctica microscope, operated under an acceleration voltage of 200 kV and equipped with Gatan K2 Summit direct electron detector. The inflammasome [8] and 26S proteasome [7] were imaged at a nominal magnification of 21,000 times. The proteasomal 19S regulatory particle (RP) [49] was imaged at a nominal magnification of 54,000 times. Cryo-EM data were collected semi-automatically using Leginon [50]. Movies comprising 36 frames per exposure were collected for the inflammasome and the 19S RP with an accumulated dose of 50 electrons/Å^2^. For the 26S proteasome, movies of 30 frames per exposure and an accumulated dose of 30 electrons/Å^2^ were obtained. The physical pixel size and the super-resolution pixel size of the inflammasome and 26S proteasome datasets were 1.72 Å and 0.86 Å, respectively. The calibrated physical pixel size of 19S RP was 0.98 Å. The defocus in data collection were set in the range of -1.0 to -3.0 μm. See S1 Fig for typical micrographs and class averages.

### *Ab initio* 3D reconstruction

To examine the effect of GTM-based clustering on *ab initio* 3D reconstruction, 117,471 RP complex particles were classified, by unsupervised GTM clustering implemented in ROME, into 1,000 classes (S2 Fig). We set the in-plane rotational search to a 2.5° increment, the translational search in the range of -4 to 4 pixels and 100 classes for MAP2D alignment. We used 234 class averages of the highest quality from the 1,000 GTM-produced classes to generate an initial model of the RP complex using e2initialmodel.py in EMAN2. In this step, 8 refinement iterations were performed with outputs of 10 initial models in search of a good one. As a control, we used the traditional method to obtain an initial model of the RP complex in EMAN2 from 10,000 raw RP complex images. These particles were first classified into 100 classes using e2refine2d.py. 12 MSA basis vectors, 5 alignment references and 8 iterations were set up for this EMAN2 procedure. 72 good classes were used to generate an initial reconstruction of RP complex by e2initialmodel.py in EMAN2. In this step, 8 refinement iterations were performed with outputs of 10 initial reconstructions. The best reconstruction was chosen for further analysis.

### Parallel implementation and code optimization

Both the MAP2D and GTM implementations in ROME are optimized specifically for Intel^®^ Xeon^®^ processors and Intel^®^ Xeon Phi^™^ coprocessors. In analogy to MAP2D in RELION, algorithm-level optimizations such as an adaptive E-M approach were adopted in ROME. In addition, the particle image stack was divided based on the unit of individual images across multiple computer nodes in the parallelization of the data flow in the MAP2D module. However, the image stack was divided based on the unit of individual image pixels in the GTM implementation. This allowed the problem to be distributed across multiple cluster nodes and attached coprocessors, while minimizing data transfer and memory requirements. Next, complex data structures were converted from arrays of structures to structures of arrays, making it easier to implement the inner loops of compute-intensive kernels with processor instructions that can be executed on multiple data elements in parallel (vectorization). Loops were also restructured so that independent threads of execution work on data elements that are near one another in memory. This ‘cache blocking’ attempts to lower the pressure that the algorithms put on the memory subsystem as the number of threads climbs, and tries to make the most effective use of the processor memory caches. To further improve the use of memory and decrease serialization overhead, memory allocation and deallocation were moved from the middle of the algorithm to the initialization phase. Finally, a single-precision data type instead of a double-precision one was used to store the image data in parts of the algorithm where the extra precision is not necessary. This treatment can improve performance by about a factor of two.

## Results

### Classification accuracy with simulated data

**Fig 2:**
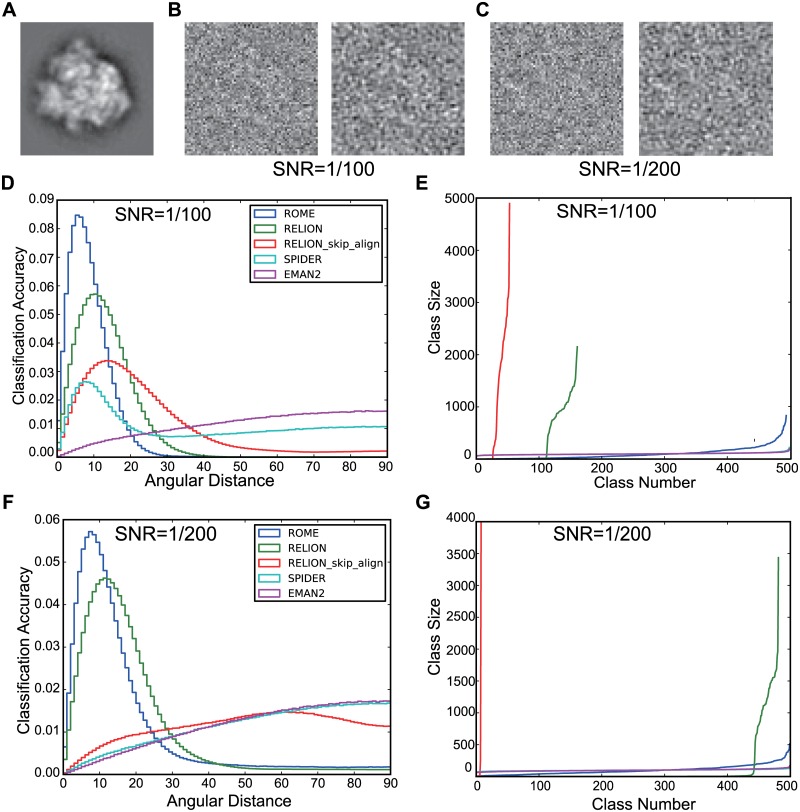
Benchmarking the performance of unsupervised clustering using simulated data. **(A)** A projection of the 70S ribosome model. **(B** and **C)** Examples of the simulated images of the 70S ribosome with SNRs of 1/100 **(B)** and 1/200 **(C)**. The right panel in **(B)** and **(C)** shows the low-pass filtered version of each simulated image. **(D** and **F)** The normalized histogram exhibits the distributions of angular distances resulting from the five classification methods that were applied to the simulated images with SNRs of 1/100 (panel **D**) and 1/200 (panel **F**). **(E** and **G)** The sizes of classes were ranked for the five classification methods with SNRs of 1/100 (panel **E**) and 1/200 (panel **G**).

To quantify the classification accuracy of our approach, we generated a series of synthetic datasets with various SNRs, each of which was composed of 50,000 simulated images as described above. Since all the original angles of the simulated data were known, the angular difference between any pair of images classified into each class (hereafter named ‘angular distances’) could be computed. We adopted the statistical behavior of the angular distances as a measure of the quality of the corresponding classes [13]. Supposing that *n* images are classified into one class, we have $\;\left(\begin{array}{c} n\\ 2 \end{array}\right)$ pairs of angular distances in this class. The histogram distribution of the angular distances from all the classes is a good way to compare the results from different algorithms [13]. A sharper, higher peak at lower angular distances in the distribution curve is expected from a better and more accurate clustering. We also used a second plot that ranks the number of particles over all classes to show the effective classes in the results. With the dataset at a SNR of 0.02, the classification accuracy of our approach is almost the same as that of SPIDER, and was better than that of both EMAN2 and RELION (S3C Fig). For the data at a SNR of 0.01, our approach yielded the sharpest and highest peak at lower angular distances in the distribution plot (Fig 2D), with a total of 495 effective classes (Fig 2E). This feature remained when the SNR was further reduced to 0.005. By contrast, the MAP2D classification implemented in RELION yielded a lower peak (Fig 2D), but only 162 effective classes (Fig 2E). The RELION results obtained with the ‘skip_align’ option were much worse than those from the standard MAP2D classification procedure (Fig 2D and 2E). Conventional PCA/K-means approaches in EMAN2 and SPIDER resulted in a much lower, wider peak at greater angular distances (Fig 2D and 2E). When the SNR was decreased to 0.005, the classification accuracies of these approaches were further reduced (Fig 2F and 2G and S3E and S3F Fig). These results suggest that a high level of noise significantly compromises the performance of data clustering by conventional PCA/K-means clustering approaches. By contrast, our GTM approach significantly improved the classification accuracy under conditions of reduced SNRs.

**Fig 3:**
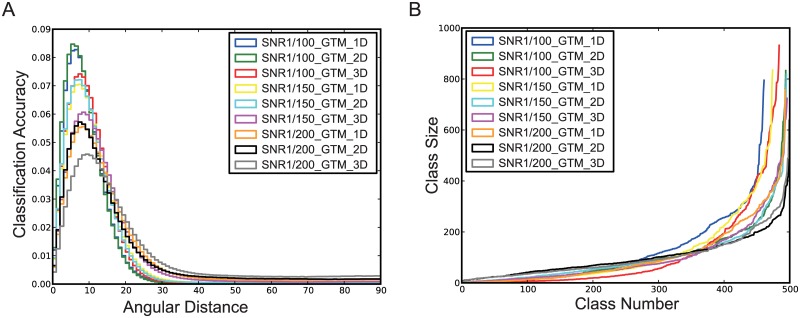
Classification accuracy with one-, two- and three-dimensional latent space in our GTM algorithm. **(A)** Normalized histograms exhibit the angular distances for the one- and two-dimensional latent space under different SNRs. **(B)** The sizes of classes are for different latent space dimensions with varying SNRs. The label ‘GTM_D’ in **(A)** and **(B)** represents the number of dimensions. GTM_1D denotes that 500 points in one dimensional latent space were sampled in the GTM algorithm. GTM_2D denotes that 100 points in one dimension and 5 points in the other dimension, a total of 500 points, were sampled by the GTM algorithm. GTM_3D denotes that 20 points in the first dimension and 5 points in each of the other two dimensions, giving a total 500 points, were sampled in the GTM algorithm.

We further examined the impact of the dimensionality of the latent space on the classification accuracy in the GTM algorithm. Using the same datasets of simulated images, we compared the measurements of the classification accuracies for the latent spaces assumed in one, two and three dimensions. The experiment with 2D latent space yielded slightly better classification accuracies than those with 1D and 3D latent space in our GTM algorithm for SNRs of 0.01, 0.0067 or 0.005 (Fig 3). This result suggests that the increased dimensionality in manifold learning does not necessarily improve classification accuracy.

We also examined the impact of several control parameters in the GTM algorithm on classification accuracy. First, there are two independent hyper-parameters in GTM: *M*_*NL*_ and *α*. Using the simulated datasets with a SNR of 1/100, we compared the measurements of the classification accuracy for different *M*_*NL*_ and *α* values. The experiment with *M*_*NL*_ = 0.8×*K*, where *K* is the class number, yielded better classification accuracy than those with smaller *M*_*NL*_ values (S4 Fig). This result suggests that an increased number of nonlinear basis functions can strengthen the classification capability of GTM. For the hyper-parameter *α*, the experiment with *α* = 0.01 yielded a better classification accuracy than those with smaller or larger *α* values (S5 Fig). This result suggests that an appropriate *α* value can penalize the ‘peaky’ elements in the weight matrix **W** to prevent overfitting and to improve the classification ability of GTM. Next, we compared the classification accuracies for different numbers of classes, namely, *K* = 100, 300 and 500. We found that the classification accuracy increases with increasing class number (S6 Fig). However, this was not the case for the other classification methods implemented in RELION and SPIDER (S6 Fig). Finally, we investigated the robustness of our GTM clustering algorithm by varying the initialization. We used different characteristic radii of the Gaussian circles and different values of *β*, as well as the outputs of PCA, for GTM initialization (S7 Fig). The results indicate that the performance of our GTM clustering is insensitive to the parameters of Gaussian circle inputs, which generally gave rise to better performance than those obtained by the PCA-based initialization (S7 Fig).

### Applications to experimental cryo-EM datasets

**Fig 4:**
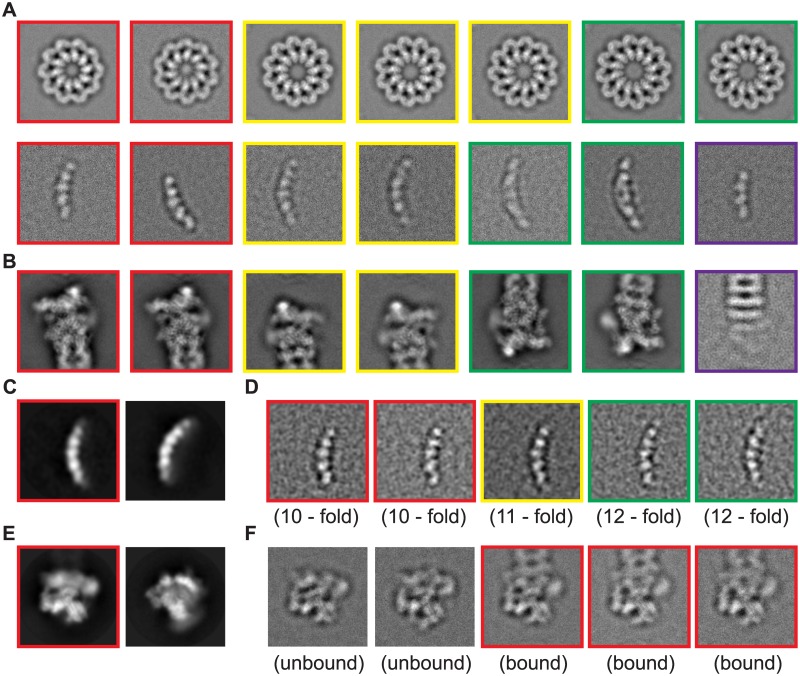
Unsupervised clustering by GTM. **(A)** Typical class averages of inflammasome particles generated by unsupervised GTM clustering in ROME. Red, yellow and green boxes indicate the top views (first row) and the side views (second row) of 10-, 11-, and 12-fold inflammasome complex, respectively. The side views of the complex structure differ by length. Besides, the purple box denotes the class average of an incomplete inflammasome complex. **(B)** Typical class averages of RP-CP sub-complexes generated by unsupervised GTM in ROME. The red or yellow boxes indicate a pair of class averages showing differences in local features corresponding to the local movement of the Rpn5 subunit of the RP-CP subcomplex [7]. The green box indicates a pair of class averages showing the movement of the Rpn1 subunit of RP-CP subcomplex [7]. The purple box labels the class average of the incomplete RP-CP subcomplex. **(C)** Typical side-view class averages of the inflammasome were initially classified using the MAP2D classifier in a reference-free manner. Two classes among 50 classes visually resemble the 11-fold inflammasome complex particles. **(D)** The class average highlighted by red box in panel (**C)** was further classified by GTM. The red boxes indicate the 11-fold inflammasome particles. The green boxes indicate the 10-fold inflammasome particles that were misclassified by MAP2D into the same class as the rest 11-fold structures. The yellow boxes indicate the 12-fold inflammasome particles that were misclassified by MAP2D into the same class as the rest of the 11-fold structures. **(E)** A 57,001-particle dataset of free RP was initially classified using the MAP2D classifier in a reference-free manner. **(F)** The class marked by the red box in panel (**E)** was further classified by GTM in ROME. Several classes of RP-CP sub-complex particles (red boxes) were found to be misclassified into this free RP class.

To demonstrate the applicability of our clustering approach, we applied our method to several experimental cryo-EM datasets (S1 Fig). First, ROME was used for an unsupervised clustering of a 17,103-particle dataset of the inflammasome complex [8]. The resulting class averages directly presented three different conformations of the complexes corresponding to 10-, 11- and 12-fold symmetry (Fig 4A and S8A Fig). For comparison, 300 classes were produced by reference-free MAP2D classification implemented in RELION [19] (S8B Fig). Second, unsupervised GTM clustering of 96,488 particles of the proteasomal regulatory particle associated with the core particle (RP-CP) sub-complex directly revealed different local features among class averages (Fig 4B) [2,7]. These local differences were verified as corresponding to distinct conformational states through further 3D reconstruction [2,7]. Our results thus indicate that unsupervised GTM clustering can sort out not only the projections of underlying structures into relatively homogenous 2D classes but can also unearth incomplete structures or junk particles, allowing the efficient *in silico* purification of single-particle datasets.

**Fig 5:**
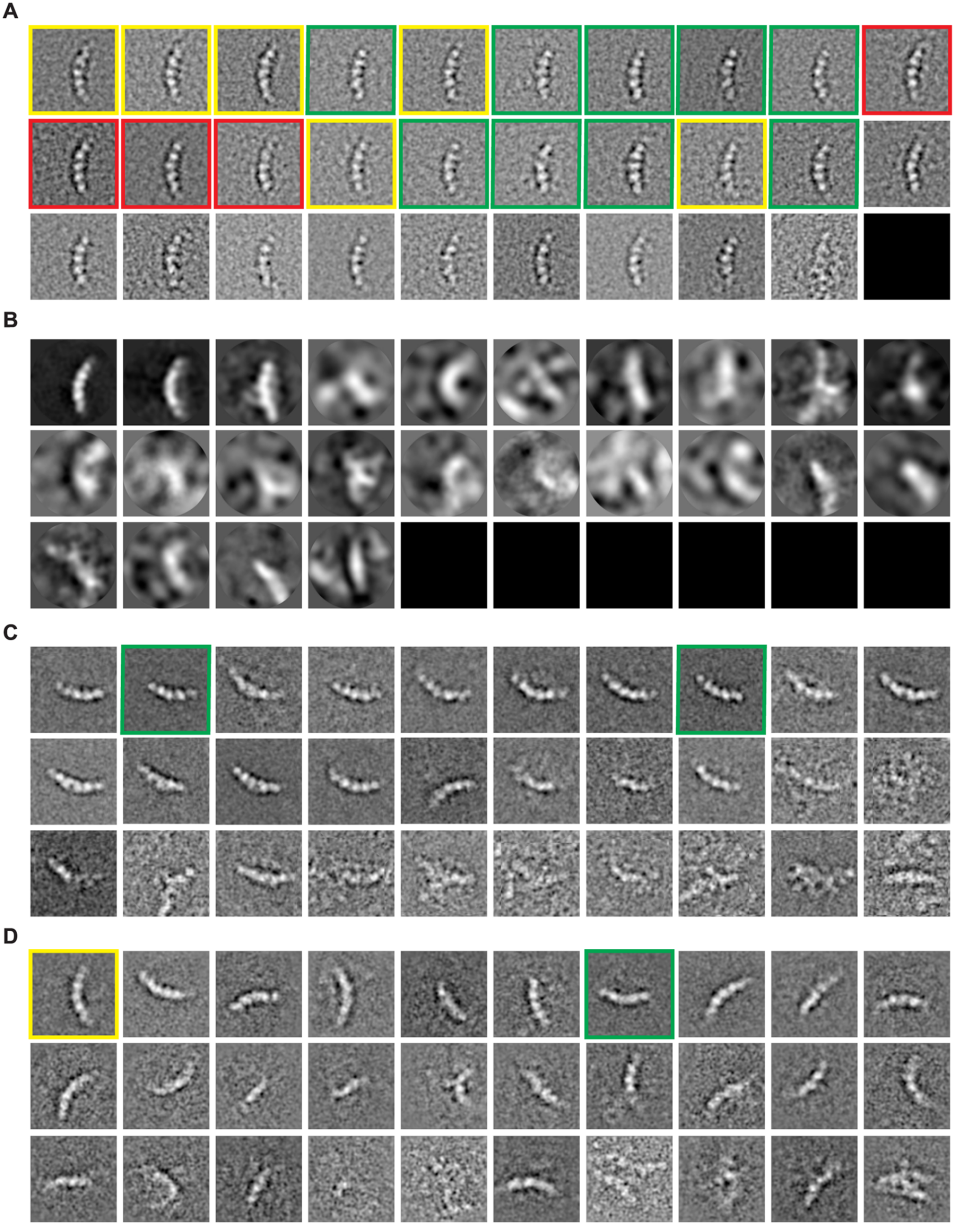
Comparison of hierarchical unsupervised clustering using ROME, RELION, SPIDER and EMAN2. **(A)** Unsupervised classification of a MAP2D-generated class into 30 sub-classes using the GTM algorithm in ROME. The red box marks the side view projection of the 11-fold inflammasome complex. The green box marks the side view projection of the 10-fold inflammasome complex, whose length is smaller than that of the others. The yellow box marks the side view projection of the 12-fold inflammasome complex, whose length is larger than that of the others. **(B)** Unsupervised classification of the same MAP2D-generated class into 30 sub-classes in RELION. The major class exhibits the side view projections of 11-fold inflammasome complex, whereas all the other classes present ‘junk’ features. **(C)** Unsupervised K-means clustering of the same MAP2D-generated class into 30 sub-classes in SPIDER. The green boxes highlight the side views of the 10-fold inflammasome complex, whose length is smaller than others. The yellow boxes label the side view projections of the 12-fold inflammasome complex, whose length is larger than that of the others. **(D)** Unsupervised K-means clustering of the same MAP2D-generated class into 30 sub-classes in EMAN2. The yellow boxes label the side view projections of the 12-fold inflammasome complex, whose length is longer than that of the others. The green boxes label the side view projections of 10-fold inflammasome complex, whose length is shorter than that of the others.

Unsupervised GTM clustering with improved accuracy can help identify hidden heterogeneity in the reference-free classes generated by other methods. For instance, after initial reference-free clustering, particle images of specific classes could be selected for deeper unsupervised clustering by GTM in a hierarchical fashion. Delicate differences between GTM-generated class averages may reveal features corresponding to distinct conformational states. To test this idea, a class of 281 particles resulting from MAP2D-based classification (Fig 4C), whose average visually resembles the side view of the 11-fold inflammasome complex, was further classified into 30 sub-classes by GTM (Fig 5A). Based on the length of the intrinsic structure, the GTM-based reference-free clustering identified the side views of 10-fold and 12-fold inflammasome complexes among the 30 sub-classes (the red boxes in Fig 4D), suggesting that the MAP2D-generated initial class is a mixture of the complexes with 10-, 11- and 12-fold symmetry. Another MAP2D-classified image group with 3961 particles, whose average visually represents a tilted view of the free 19S regulatory particle (RP) that is not associated with a 20S core particle (CP), was further classified by GTM (Fig 4E). This deeper clustering identified 448 single-particle images (11.3%) of RP-CP subcomplexes that were misclassified into the dataset of free RP (Fig 4F).

Fig 5 presents a further comparison with other algorithms including MAP2D and K-means clustering, which suggests that our GTM outperforms those algorithms in unambiguously identifying hidden heterogeneity with the hierarchical clustering strategy. In this comparison**,** we selected a MAP2D-classified image group comprising 281 side views of the inflammasome complexes (red boxes in Fig 4C), which was further classified into 30 classes by unsupervised GTM clustering in ROME, by reference-free MAP2D classification in RELION, and by K-means clustering in SPIDER and EMAN2. The sub-classes resulting from the GTM algorithm all showed clear 2D structural features, indicating a mixture of the side views of 10-, 11- and 12-fold complexes in this dataset, as manifested by their length variation (green box and yellow box in Fig 5A). By contrast, only two meaningful classes were obtained by RELION, while the other sub-classes were either featureless or empty (Fig 5B). The results from SPIDER and EMAN2 appear to be improved over RELION. However, there were still more than 10 sub-classes in both cases that were either featureless or presented aberrant features likely resulting from mis-classification. Our comparative studies suggest that, unlike our GTM-based clustering, other unsupervised clustering approaches, such as MAP2D, PCA and K-means, are either less efficient or ineffective for directly detecting subtle structural variations using the hierarchical clustering strategy.

### GTM-based clustering improves *ab initio* reconstruction

**Fig 6:**
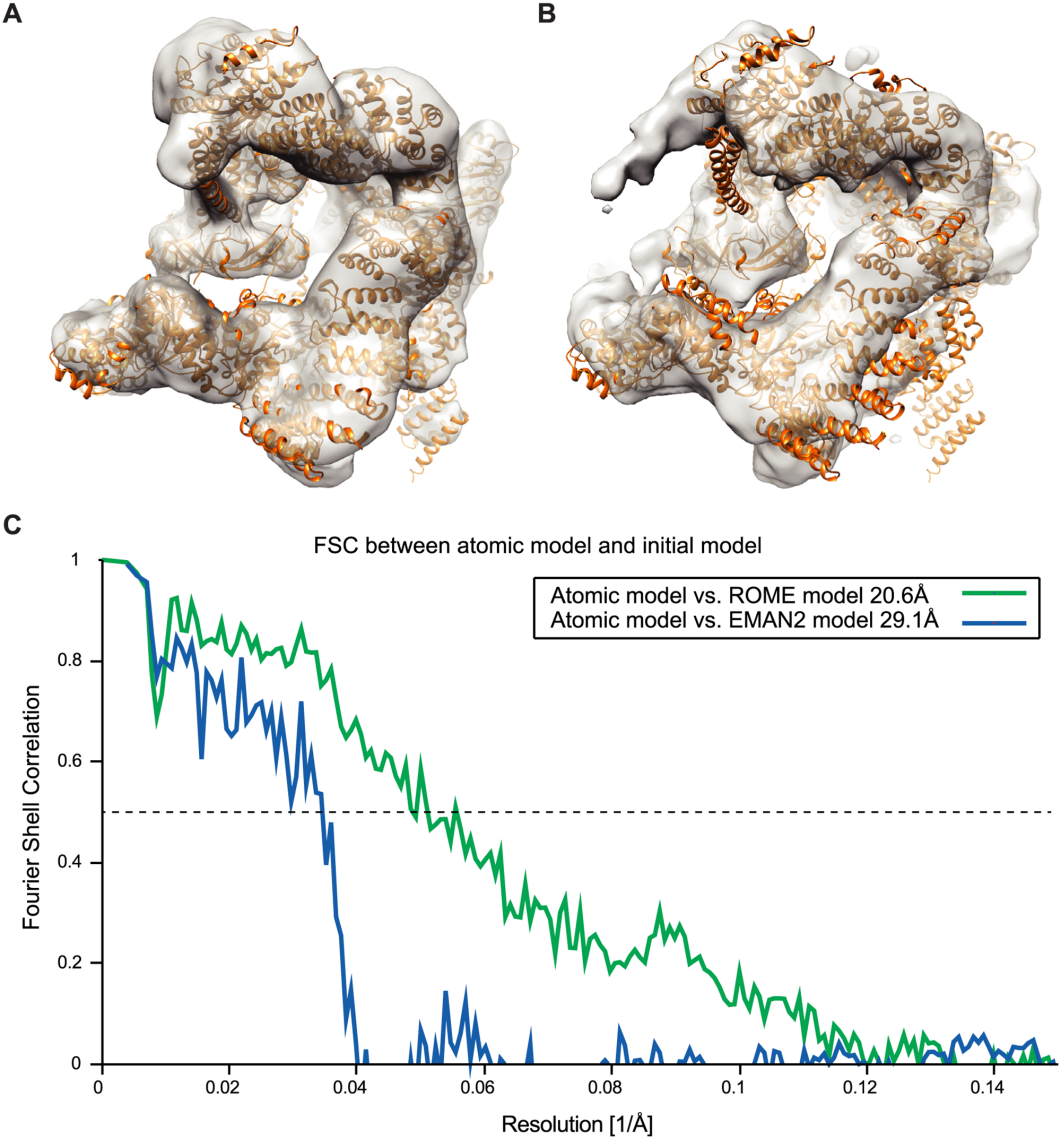
Initial 3D reconstruction from the reference-free class averages of ROME and EMAN2. **(A)** The initial reconstruction calculated by the ROME-generated class averages is superimposed with the atomic model of free RP shown in a ribbon representation, suggesting that they are highly compatible with each other. **(B)** The initial reconstruction calculated by the EMAN2-generated class averages is superimposed over the atomic model of free RP shown in a ribbon representation. A substantial part of the atomic model is outside of the density of the initial reconstruction, suggesting poor map quality and a large reconstruction error. **(C)** FSC curves between the RP atomic model and the initial reconstructions generated by ROME- and EMAN2-based class averages.

To demonstrate the clustering capacity of our approach, 117,471 particles of the free RP complex were classified into 1,000 classes by GTM implemented in ROME in an unsupervised manner. The computation was completed in 228 minutes with 30 rounds of E-M optimization on a computing cluster comprising 512 Intel Xeon E5 CPU cores (S1 Table) and 858 effective classes were obtained (S2 Fig). We used 234 class averages of the highest quality from the 1,000 unsupervised classes to generate an initial 3D reconstruction of the RP complex in EMAN2 (Fig 6A, S2 Fig). As a control, we used the conventional multivariate data analysis (MDA) and K-means clustering to obtain an initial reconstruction of RP complex in EMAN2 from 10,000 raw images of the RP complex (Fig 6B). The FSC curves between the atomic model and the two initial reconstructions using a 0.5-cutoff suggest that the resolution of the initial model using GTM-based class averages was 20.6 Å, which was significantly higher than the resolution, 29.1 Å, of the other initial reconstruction obtained by the conventional approach (Fig 6C). Next, we performed rigid-body fitting of the atomic model of the free RP into the two initial reconstructions. The atomic model fits into the GTM-based initial reconstruction with an excellent agreement (Fig 6A). By contrast, considerable elements in the atomic model were out of the density of the initial reconstruction obtained by the conventional approach (Fig 6B). The contrasting results show that the initial reconstruction built upon our GTM clustering method is of significantly improved quality compared with the conventional one. Taken together, these results suggest that the increased class number and improved classification accuracy of our unsupervised GTM clustering gives rise to a marked improvement of *ab initio* 3D reconstruction with an affordable computational cost.

### Optimization of computational performance and efficiency

**Fig 7:**
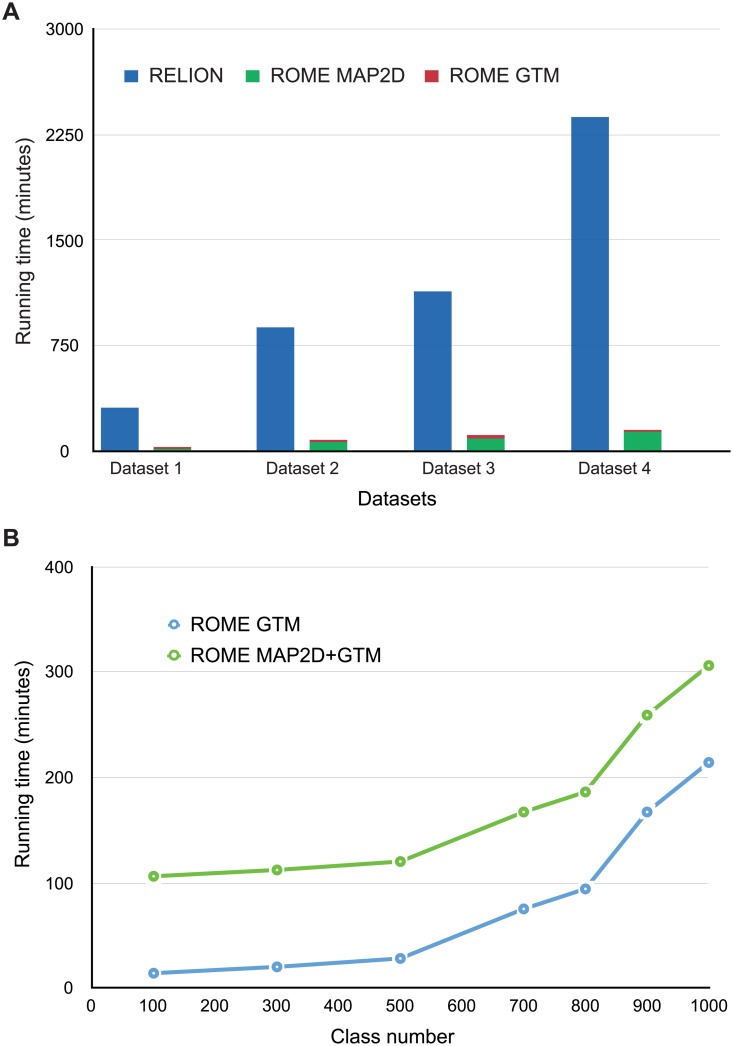
Performance evaluation of unsupervised clustering with ROME. **(A)** Performance of unsupervised single-particle clustering in ROME versus RELION using different datasets. Unsupervised 2D classification into 300 classes using both software programs were performed on four experimental datasets: Dataset1 refers to the 16,306-particle dataset of the inflammasome with 250×250 box size; dataset2 refers to the 35,407-particle dataset of the free RP complex with 160×160 box size; dataset3 refers to the 96,488-particle dataset of the RP-CP complex with 160×160 box size; dataset4 refers to the 57,001-particle dataset of the free RP complex with 180×180 box size. MAP2D alignment in ROME and GTM clustering for 300 classes wes also performed. The blue, green, and red histograms represent the running time of RELION, MAP2D in ROME, and GTM in ROME, respectively. For more comparison, see S9 Fig and S1 Table. **(B)** The 96,488-particle dataset of the RP-CP subcomplex was used to test the performance of GTM in ROME (blue dots). The green dots represent the total running time including both the MAP2D alignment and GTM clustering in ROME. The running time was polynomially related to the number of classes.

Our implementation of the GTM-based clustering algorithm in the ROME software has been optimized for modern HPC hardware, such as Intel^®^ MIC architecture used in the Intel Xeon Phi^™^ Knights Corner and Knight Landing processors. Our modernized code in ROME outperforms the existing software in both speed and magnitude. First, we compared the computational performance between ROME and RELION using several datasets comprising different numbers of particles. It took 143 minutes for ROME to classify 57,001 particles of RP into 300 unsupervised classes on a computing cluster comprising 512 Intel Xeon E5 CPU cores (Fig 7A). By contrast, it took ~40 hours for RELION to classify the same dataset into 300 unsupervised classes on the same cluster. In all cases, the E-M algorithm was run for 30 iterations. Thus, under the same circumstances, ROME was about 10–20 times faster than RELION in unsupervised 2D classification. Second, a 96,488-particle dataset of the RP complex was used to benchmark the performance of ROME in unsupervised clustering with different class numbers on the 512-core computing cluster (Fig 7B). With increasing class numbers from 100 to 1,000, the running time of GTM clustering was increased in a polynomial trend from 14 to 214 minutes. Specifically, ROME completed the unsupervised clustering of 1,000 classes in 306 minutes with 30 iterations of expectation-maximization (S1 Table), whereas RELION did not complete the same number of iterations of optimization in a week on the same hardware condition. Furthermore, we also used this dataset to test reference-free classification at the 1,000-class level by MSA and K-means clustering in SPIDER [1,20]. Unfortunately, the program kernel crashed in the reference-free alignment step.

## Discussion

Unsupervised data clustering plays an important role in high-resolution structure determination by single-particle cryo-EM [1]. In this study, we introduced a GTM-based unsupervised clustering method incorporating MAP2D-based image alignment, and implemented this approach in the open-source software ROME. One of the key findings of this study is that GTM-based clustering is more tolerant against noise than the other approaches we compared, including maximum-likelihood, PCA and K-means algorithms. This hallmark provides a path towards improved classification accuracy at lower SNRs. Our GTM algorithm built the CTF correction into its mathematical kernel so that the clustering would not be affected by the variation of the defocus value inherent in cryo-EM data. Our software uses the E-M algorithm to maximize the regularized log-likelihood function in statistical nonlinear mapping between the latent and data spaces, which only guarantees the solution of a local optimum. However, the random initialization of the nonlinear mapping ensures the unsupervised nature of data clustering. The class averaging expression under our GTM scheme resembles the Wiener-type filter and gives rise to CTF-corrected, probability-weighted class averages, which inherits the advantage and traits of the maximum-likelihood based approaches [1,25,26].

Utility strategies of unsupervised single-particle clustering by GTM can be highly versatile, problem-oriented, and user-controlled. In a typical scenario, one can pursue a strategy in which all particles are aligned and partitioned into unsupervised classes in single runs of E-M optimization. This gives a glimpse of typical reference-free projection structures of the biomolecules imaged and allows the removal of apparent ‘junks’. In other scenarios, particles in specific classes can be further partitioned into deeper sub-classes in a hierarchical fashion. This may allow the user to inspect subtle structural variations within a class to analyze the potential structural heterogeneity within the dataset. This hierarchical clustering strategy can be further interleaved with 3D classification to help verify the quality of 3D classification and clean up ‘junk’ or poor quality images from 3D classes [2,7,8].

In summary, we developed a statistical manifold learning-based approach for unsupervised single-particle clustering in a massively parallel manner. When optimized for Intel^®^ Xeon^®^ processors and Intel^®^ Xeon Phi^™^ coprocessors, it exhibited an unprecedented power in obtaining thousands of reference-free class averages with significantly improved clustering accuracy in a massively parallel fashion. Importantly, a greater number of high-quality unsupervised 2D class averages can lead to a more reliable *ab initio* 3D reconstruction. Practical applications of the algorithm to several experimental datasets demonstrated that unsupervised GTM clustering in ROME is highly effective in distinguishing structural heterogeneity among single-particle datasets. This can in turn, when used iteratively with 3D classification, assist in improving the structural homogeneity of cryo-EM datasets. Our approach has already been used in several studies of high-resolution structural determination from heterogeneous samples [7,8,49].

## Supporting information

S1 Fig Typical results of unsupervised clustering by GTM on three experimental cryo-EM datasets. (**A**) A typical cryo-EM micrograph of the inflammasome. The white boxes mark the particles picked from the micrograph that contributed to the dataset used in testing of our GTM algorithm implemented in ROME. (**B**) Typical reference-free 2D class averages of the 10-fold, 11-fold, and 12-fold inflammasome complex obtained by GTM-based clustering following MAP2D-based image alignment. (**C**) A typical cryo-EM micrograph of the proteasome. We boxed half of the holoenzyme, including half of the CP, in complex with a complete RP, named the RP-CP subcomplex (white boxes). (**D**) Typical reference-free 2D class averages of the RP-CP subcomplex obtained by GTM-based clustering following MAP2D-based image alignment. (**E**) A typical cryo-EM micrograph of the human RP proteasome. We boxed all particles, including the free RP complex and RP-CP subcomplex, whose box center is focused on that of RP (white box). (**F**) Typical reference-free 2D class averages of the RP complex obtained by GTM-based clustering following MAP2D-based image alignment.
(PDF)

S2 Fig Unsupervised clustering of the RP dataset by ROME. 117,471 particles of the RP complex were classified into 1,000 classes by the unsupervised GTM implemented in ROME. 858 class averages were not blank. These classes comprised different views of the RP complex.
(PDF)

S3 Fig Comparison of classification results of simulated data at different noise levels. (**A**, **B**) Examples of the corresponding simulated images of the 70S ribosome with SNRs of 1/50 (**A**) and 1/150 (**B**), respectively. The right panel in (**A**) and (**B**) shows the low-pass filtered version of each simulated image. **(C**, **E)** Normalized histograms show the distributions of angular distances resulting from the five classification methods that were applied to the simulated images with SNRs of 1/50 (panel **C**) and 1/150 (panel **E**). (**D**, **F**) The sizes of the classes were ranked with respect to the five classification methods for SNRs of 1/50 (panel **C**) and 1/150 (panel **E**).
(PDF)

S4 Fig Impact of different values of the hyper-parameters *M*_*NL*_ in GTM on the unsupervised classification of the simulated data with an SNR of 1/100. *M*_*NL*_ was set as 0.8×*K*, 0.5×*K* and 0.2×*K*, where *K* is the class number. (**A, C, E**) Normalized histograms showing the distributions of angular distances corresponding to different *M*_*NL*_ values. The images were classified into 100 (panel **A**), 300 (panel **C**), and 500 (panel **E**) classes. (**B, D, F**) The sizes of the classes were ranked for different *M*_*NL*_ values with *K* = 100 (panel **B**), 300 (panel **D**), and 500 (panel **F**).
(PDF)

S5 Fig Impact of different values of the hyper-parameter *α* in GTM on the unsupervised classification of the simulated data with an SNR of 1/100. *α* is set as 0.0001, 0.01, 1.0 and 100. (**A, C, E**) Normalized histograms showing the distributions of angular distances corresponding to different *α* values. The images were classified into 100 (panel **A**), 300 (panel **C**), and 500 (panel **E**) classes. (**B, D, F**) The sizes of the classes were ranked for different *α* values with *K* = 100 (panel **B**), 300 (panel **D**), and 500 (panel **F**).
(PDF)

S6 Fig Impact of different class numbers *K* on the unsupervised classification of the simulated data with an SNR of 1/100. (**A**, **C**, **E**) Normalized histograms showing the distributions of angular distances resulting from the five classification methods. The images were classified into 100 (panel **A**), 300 (panel **C**), and 500 (panel **E**) classes. (**B**, **D**, **F**) The sizes of the classes were ranked for the five classification methods for *K* = 100 (panel **B**), 300 (panel **D**), and 500 (panel **F**).
(PDF)

S7 Fig Impact of different initialization strategies in GTM on the unsupervised classification of the simulated data with an SNR of 1/100 into 500 classes. (**A**, **B**, **C**) Examples of the Gaussian distribution circles corresponding to characteristic radii with 0.1×width (panel **A**), 0.25×width (panel **B**) and 0.4×width (panel **C**). (**D**) A normalized histogram showing the distributions of angular distances resulting from different initialization of the weight matrix **W.** GCI refers to the use of the Gaussian distribution circles to initialize the weight matrix **W.** PCAI refers to the use of the PCA to initialize the weight matrix **W.** (**E**) The sizes of the classes were ranked for the cases from different initializing the weight matrices **W**. (**F**) A normalized histogram showing the distributions of angular distances resulting from different initialization of *β*. *β* was set to 1, 0.01 and 100. PCAI refers to the use the PCA to initialize *β*. (**G**) The sizes of the classes were ranked for different initializing *β* values.
(PDF)

S8 Fig Comparison of unsupervised classification of the inflammasome dataset by GTM and MAP2D. 17,103 particles of inflammasome were classified into 300 reference-free classes. Only classes whose particle numbers were greater than 9 were shown. (**A**) Unsupervised clustering using GTM in ROME. Among 300 classes, 49 classes showed the various views of inflammasome complexes with different symmetry. (**B**) Unsupervised classification using the MAP2D procedure in RELION. Only 20 classes exhibited views of inflammasome complexes with different symmetry. The MAP2D in RELION generated significantly fewer effective classes than did the GTM in ROME, indicating that GTM is more efficient for distinguishing structural differences.
(PDF)

S9 Fig Performance evaluation of MAP2D-based classification algorithms implemented in ROME and RELION. A 96,488-particle dataset of the RP-CP subcomplex particles with a box size of 180×180 pixels was used to test the performance of MAP2D in RELION 1.3 and MAP2D in ROME 1.0. When increasing the class number from 30 to 300, the running time of MAP2D classification in ROME followed a polynomial behavior from 92 to 537 minutes (blue histogram).
(PDF)

S10 Fig Typical weighted loss function of the unsupervised clustering on six datasets using GTM. (**A-D**) The weighted loss function at each iteration for datasets 1 (panel **A**), 2 (panel **B**), 3 (panel **C**) and 4 (panel **D**). The four datasets were classified into 300 classes by GTM. (**E**) The dataset 5 was classified into 1,000 classes. The results of the weighted loss function at each iteration are shown. (**F**) The simulated data were classified into 100, 300 and 500 classes. The results of the weighted loss function at each iteration are shown.
(PDF)

S1 Table Summary of computational run time of 2D classification based on ROME and RELION for different classification experiments. All tests were executed on the same computing cluster (32 nodes) consisting of 512 Intel Xeon E5 CPU cores. Based on the convergence criteria using the weighted loss function, all datasets converged within 25 iterations (see S10 Fig). RELION in the table refers to the unsupervised MAP2D-based classification in RELION 1.3. ROME_MAP in the table refers to the unsupervised 2D classification based on MAP2D methods implemented in ROME. ROME_SML in the table refers to the unsupervised 2D classification based on the GTM method implemented in ROME. In all cases, the E-M algorithm was run for 30 iterations.
(PDF)
