# Supplementary figures and images for: Massively parallel unsupervised single-particle cryo-EM data clustering via statistical manifold learning
Source: PLoS One. 2017 Aug 7;12(8):e0182130. doi: 10.1371/journal.pone.0182130 (PMC5546606; doi:10.1371/journal.pone.0182130)

**A**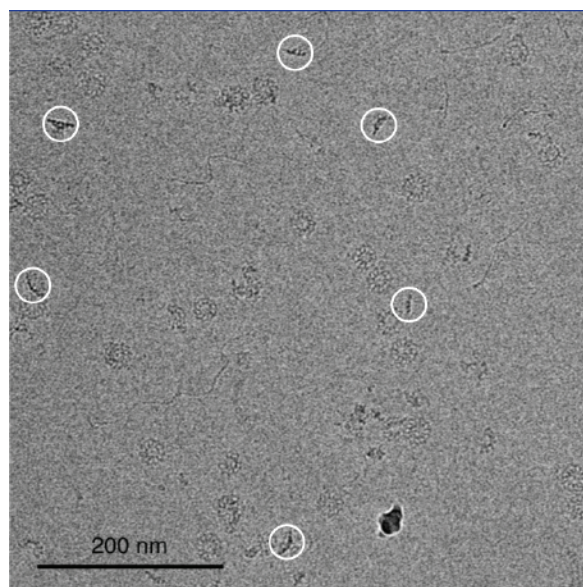**B**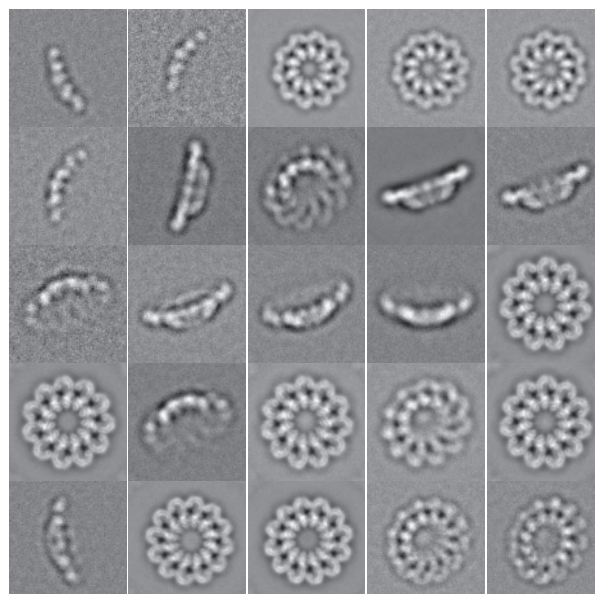**C**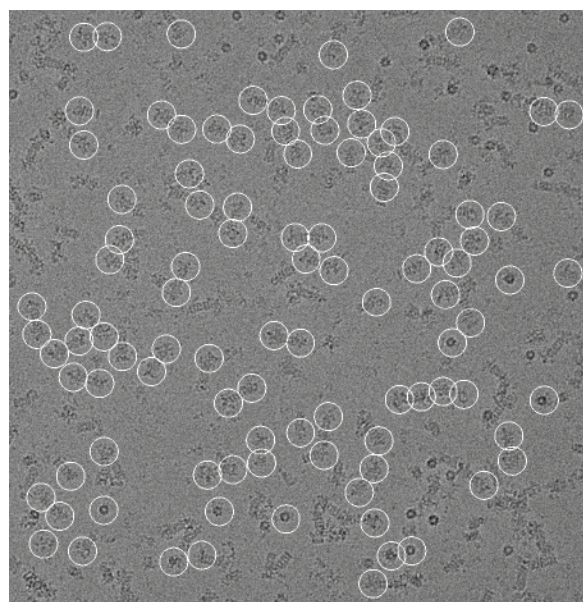**D**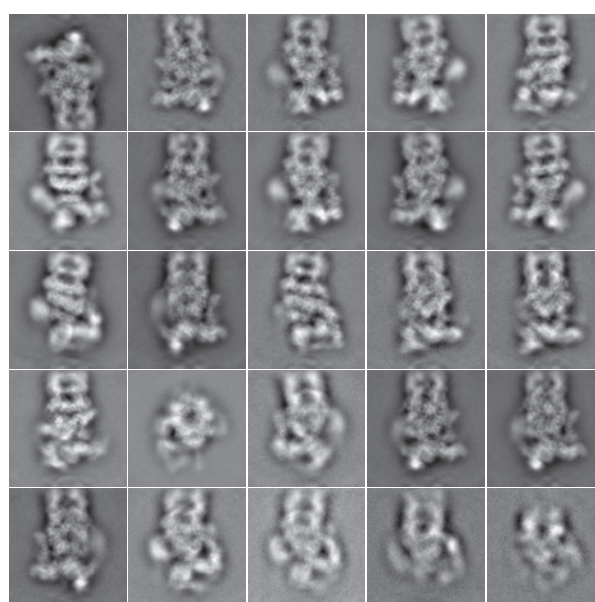**E**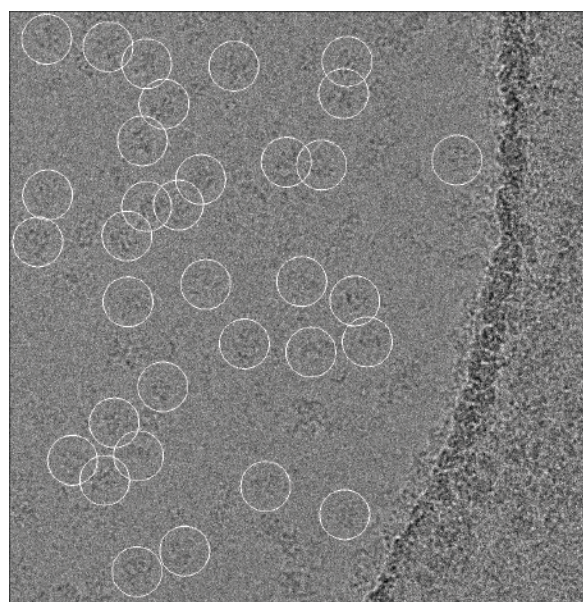**F**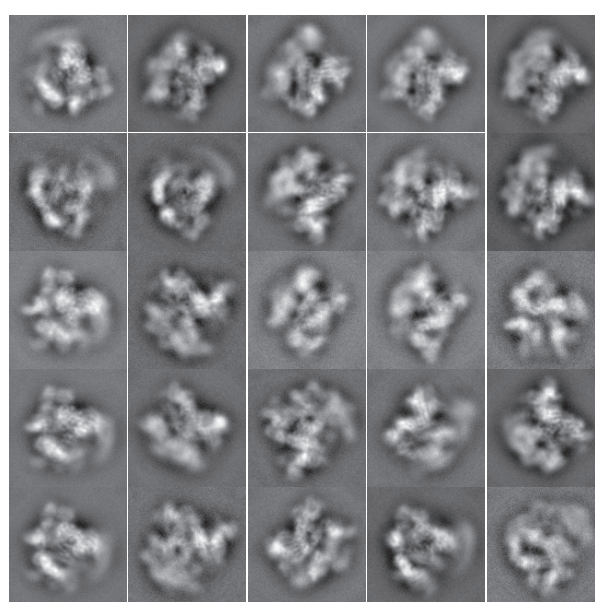

Supplement: S1 Fig — (A) A typical cryo-EM micrograph of the inflammasome. The white boxes mark the particles picked from the micrograph that contributed to the dataset used in testing of our GTM algorithm implemented in ROME. (B) Typical reference-free 2D class averages of the 10-fold, 11-fold, and 12-fold inflammasome complex obtained by GTM-based clustering following MAP2D-based image alignment. (C) A typical cryo-EM micrograph of the proteasome. We boxed half of the holoenzyme, including half of the CP, in complex with a complete RP, named the RP-CP subcomplex (white boxes). (D) Typical reference-free 2D class averages of the RP-CP subcomplex obtained by GTM-based clustering following MAP2D-based image alignment. (E) A typical cryo-EM micrograph of the human RP proteasome. We boxed all particles, including the free RP complex and RP-CP subcomplex, whose box center is focused on that of RP (white box). (F) Typical reference-free 2D class averages of the RP complex obtained by GTM-based clustering following MAP2D-based image alignment. (PDF) [file pone.0182130.s001.pdf]

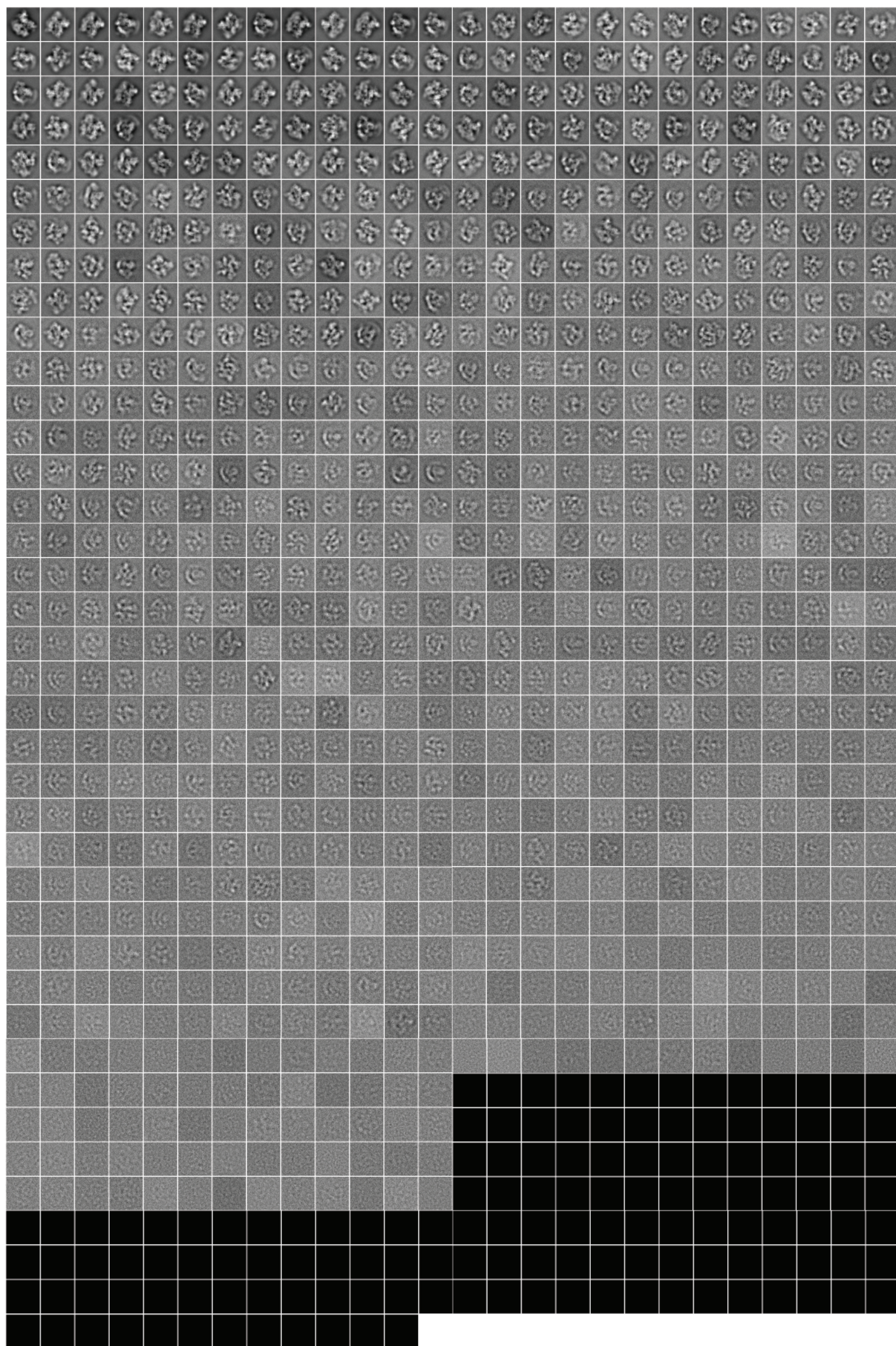

Supplement: S2 Fig — 117,471 particles of the RP complex were classified into 1,000 classes by the unsupervised GTM implemented in ROME. 858 class averages were not blank. These classes comprised different views of the RP complex. (PDF) [file pone.0182130.s002.pdf]

**A**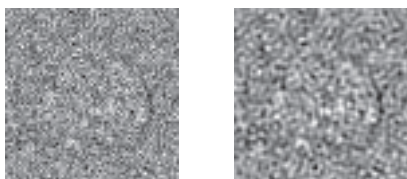

SNR=1/50

**B**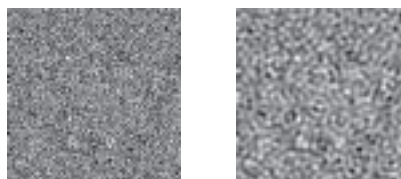

SNR=1/150

**C**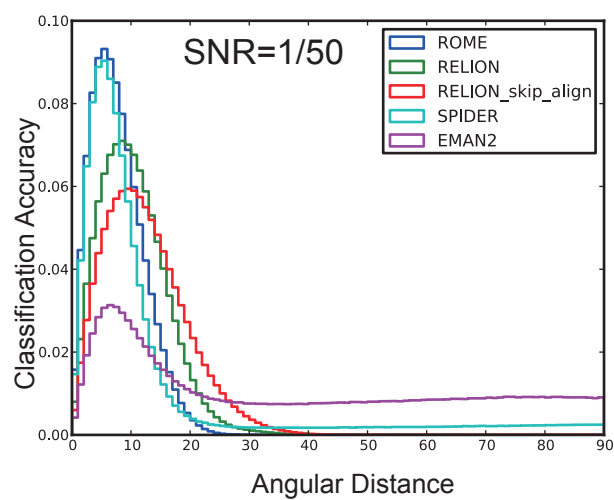**D**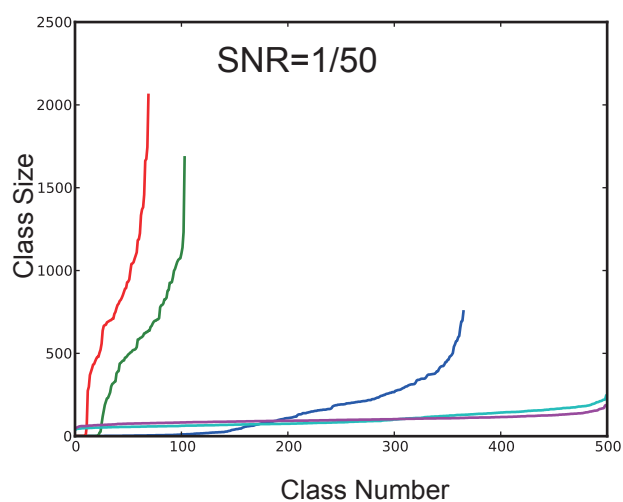**E**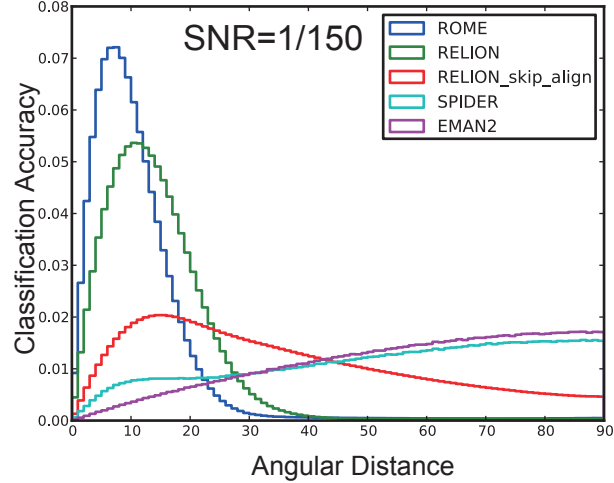**F**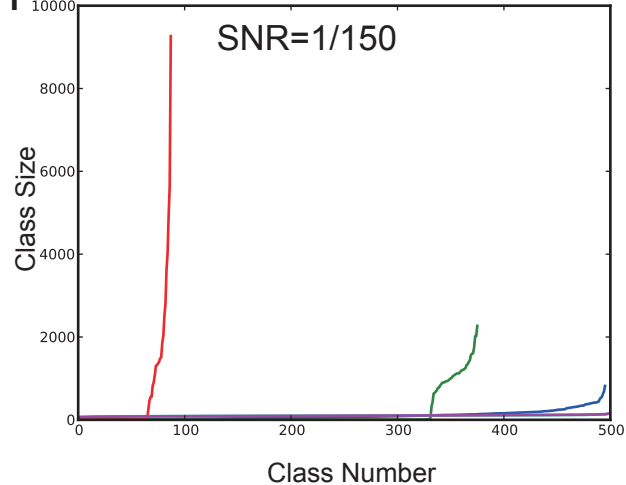

Supplement: S3 Fig — (A, B) Examples of the corresponding simulated images of the 70S ribosome with SNRs of 1/50 (A) and 1/150 (B), respectively. The right panel in (A) and (B) shows the low-pass filtered version of each simulated image. (C, E) Normalized histograms show the distributions of angular distances resulting from the five classification methods that were applied to the simulated images with SNRs of 1/50 (panel C) and 1/150 (panel E). (D, F) The sizes of the classes were ranked with respect to the five classification methods for SNRs of 1/50 (panel C) and 1/150 (panel E). (PDF) [file pone.0182130.s003.pdf]

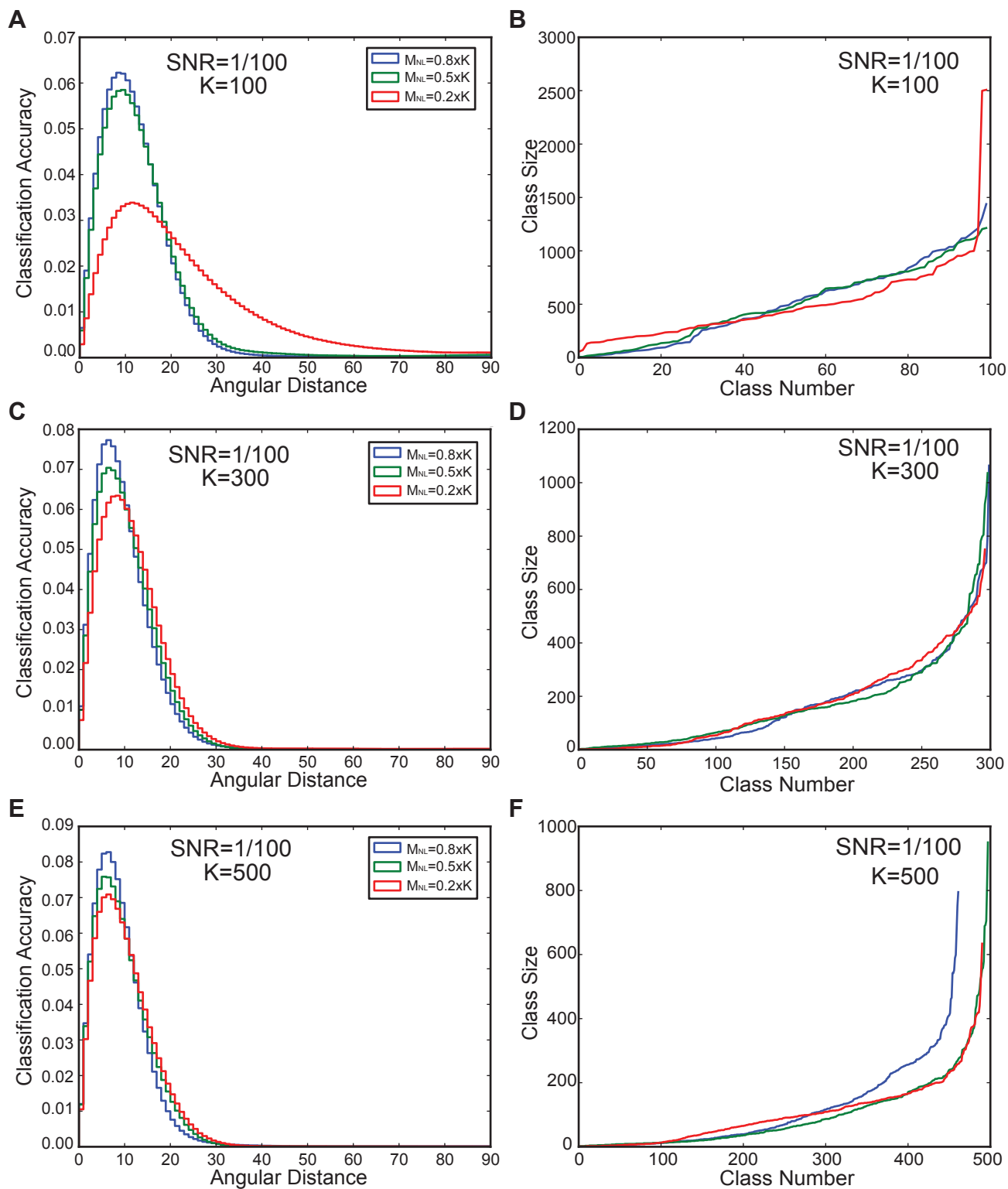

Supplement: S4 Fig — MNL was set as 0.8×K, 0.5×K and 0.2×K, where K is the class number. (A, C, E) Normalized histograms showing the distributions of angular distances corresponding to different MNL values. The images were classified into 100 (panel A), 300 (panel C), and 500 (panel E) classes. (B, D, F) The sizes of the classes were ranked for different MNL values with K = 100 (panel B), 300 (panel D), and 500 (panel F). (PDF) [file pone.0182130.s004.pdf]

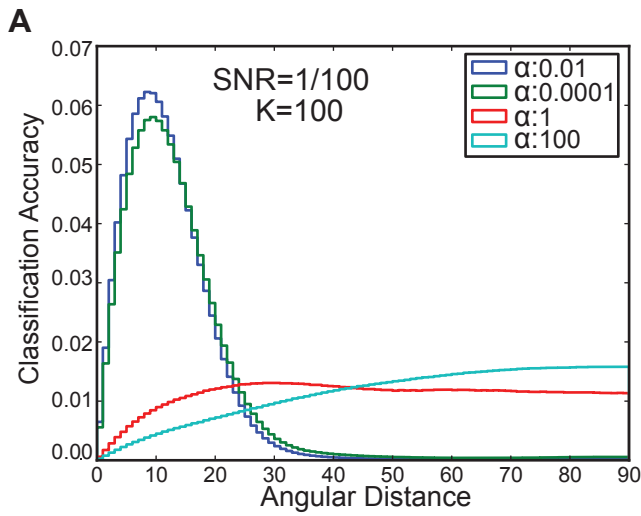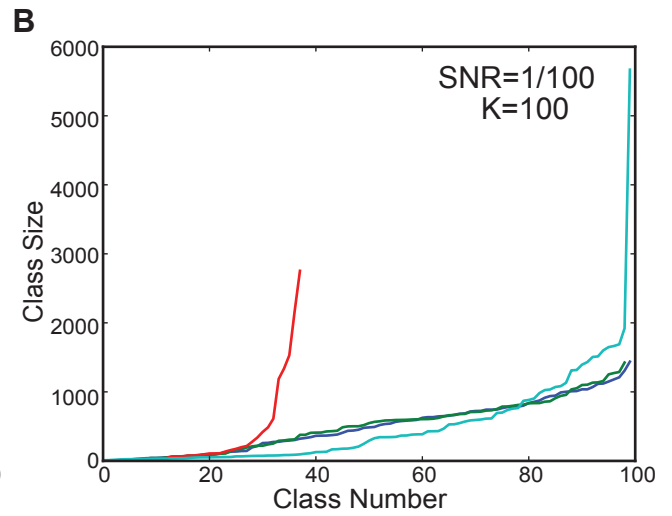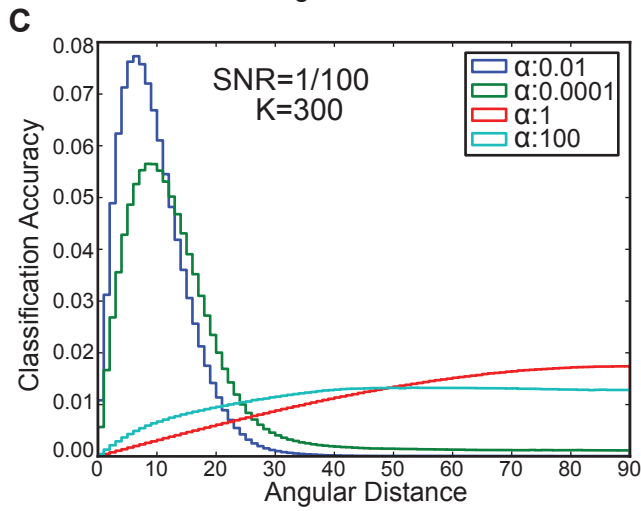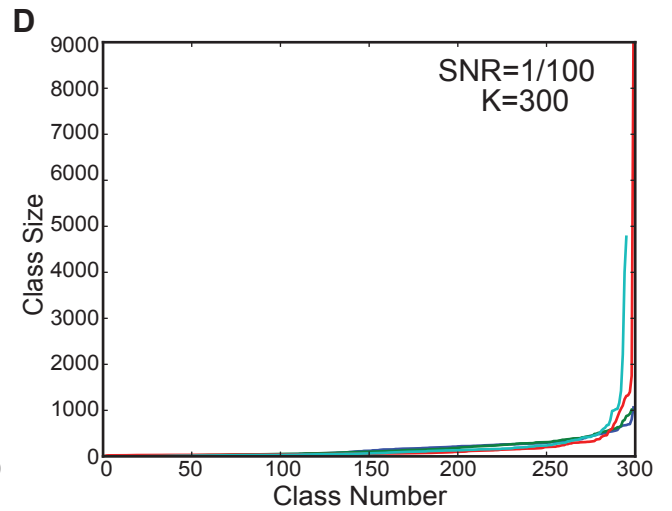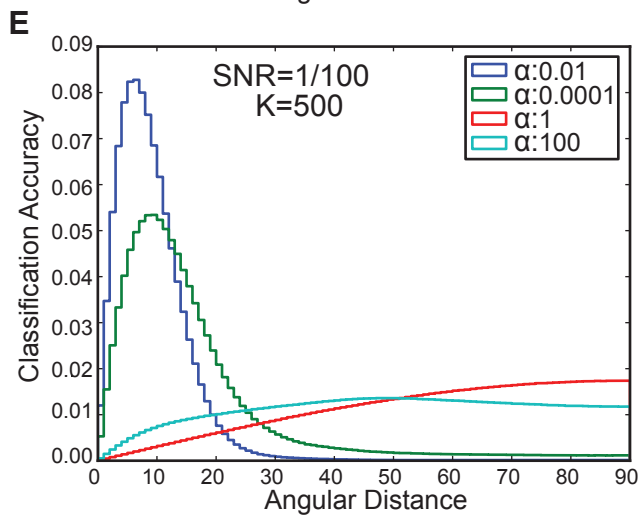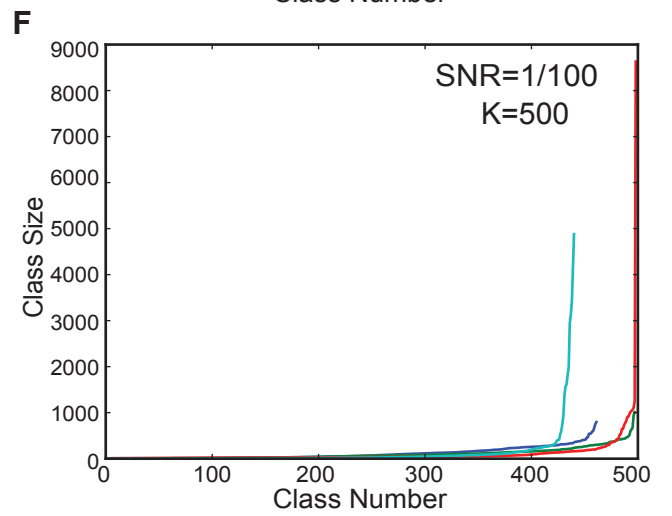

Supplement: S5 Fig — α is set as 0.0001, 0.01, 1.0 and 100. (A, C, E) Normalized histograms showing the distributions of angular distances corresponding to different α values. The images were classified into 100 (panel A), 300 (panel C), and 500 (panel E) classes. (B, D, F) The sizes of the classes were ranked for different α values with K = 100 (panel B), 300 (panel D), and 500 (panel F). (PDF) [file pone.0182130.s005.pdf]

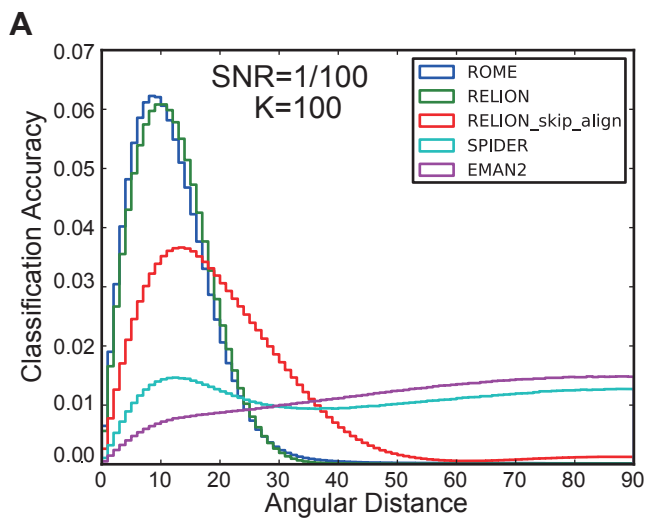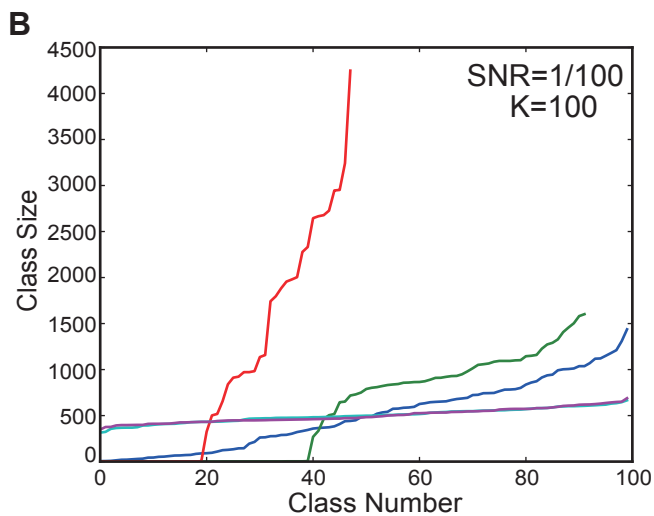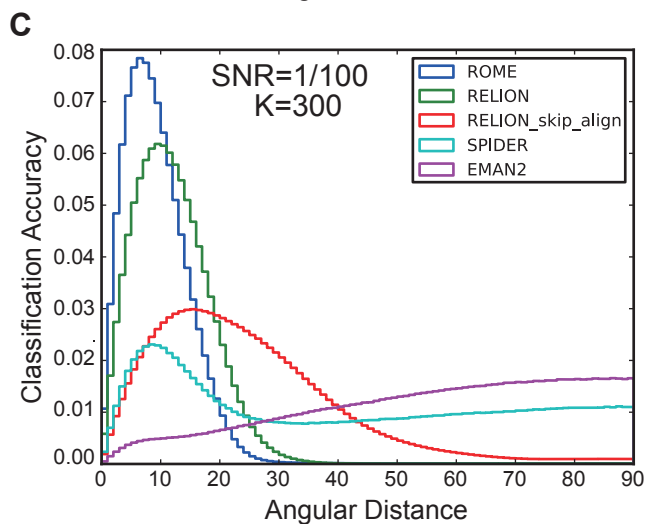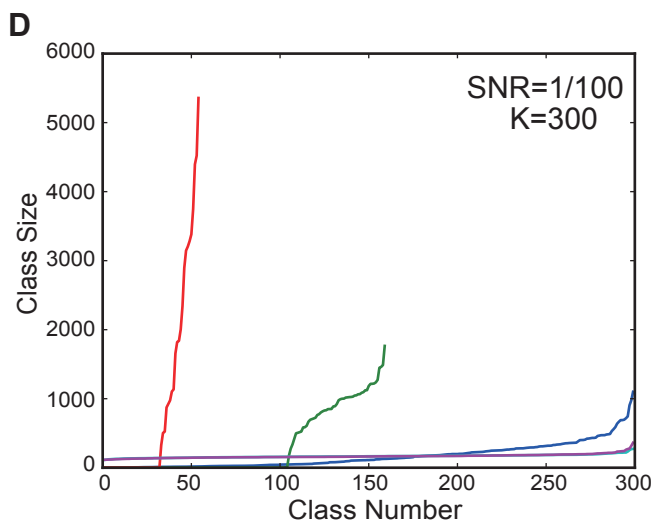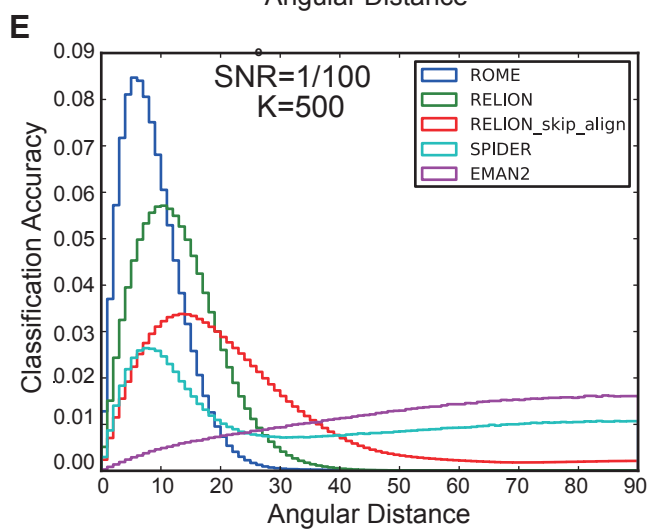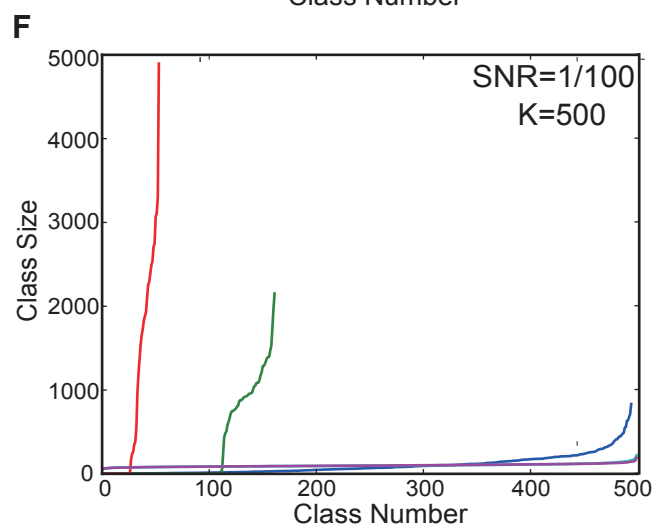

Supplement: S6 Fig — (A, C, E) Normalized histograms showing the distributions of angular distances resulting from the five classification methods. The images were classified into 100 (panel A), 300 (panel C), and 500 (panel E) classes. (B, D, F) The sizes of the classes were ranked for the five classification methods for K = 100 (panel B), 300 (panel D), and 500 (panel F). (PDF) [file pone.0182130.s006.pdf]

**A**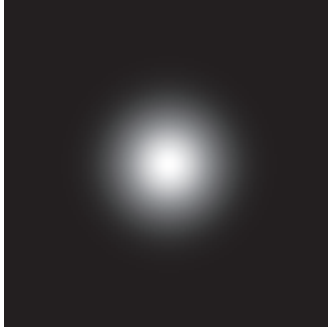

Radius=0.1xwidth

**B**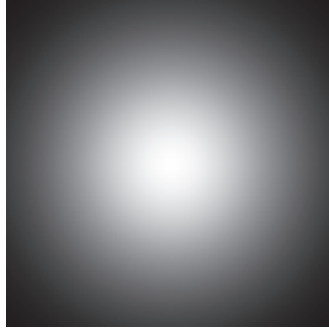

Radius=0.25xwidth

**C**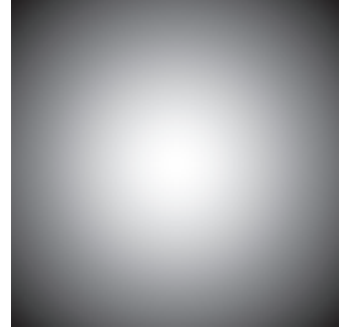

Radius=0.4xwidth

**D**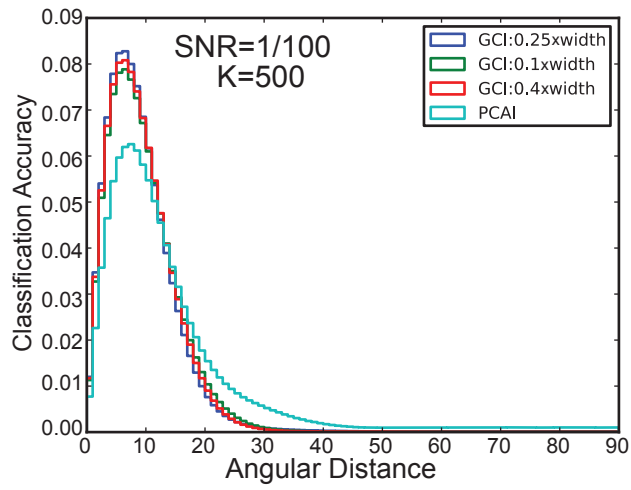**E**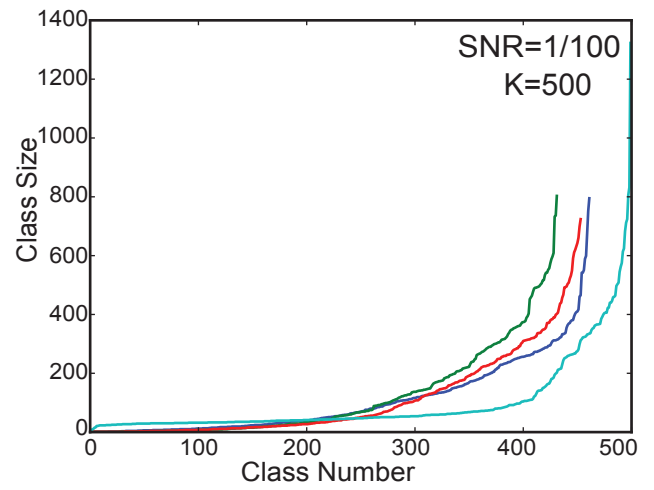**F**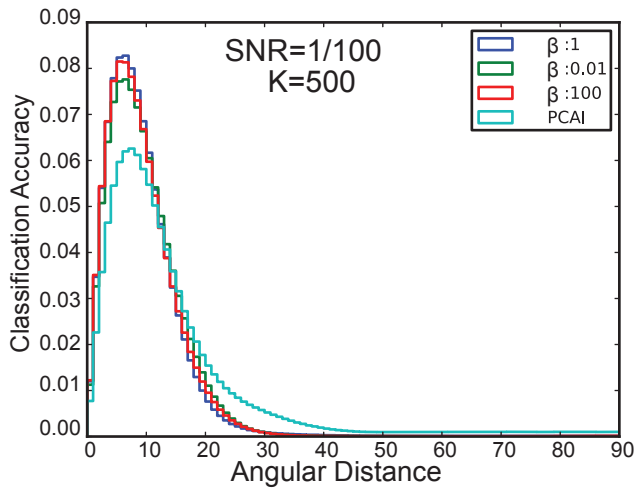**G**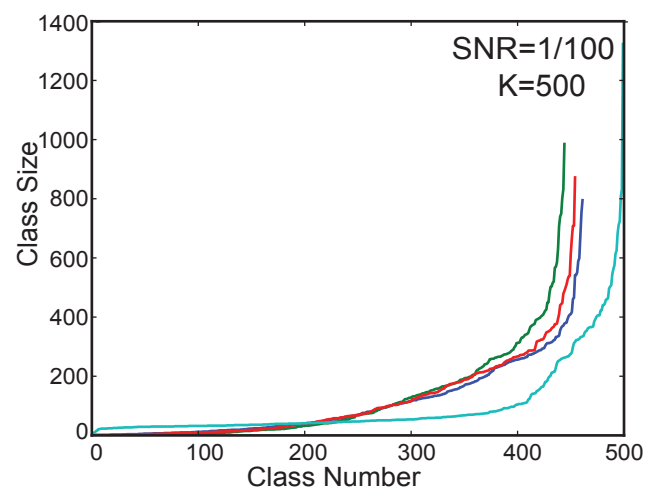

Supplement: S7 Fig — (A, B, C) Examples of the Gaussian distribution circles corresponding to characteristic radii with 0.1×width (panel A), 0.25×width (panel B) and 0.4×width (panel C). (D) A normalized histogram showing the distributions of angular distances resulting from different initialization of the weight matrix W. GCI refers to the use of the Gaussian distribution circles to initialize the weight matrix W. PCAI refers to the use of the PCA to initialize the weight matrix W. (E) The sizes of the classes were ranked for the cases from different initializing the weight matrices W. (F) A normalized histogram showing the distributions of angular distances resulting from different initialization of β. β was set to 1, 0.01 and 100. PCAI refers to the use the PCA to initialize β. (G) The sizes of the classes were ranked for different initializing β values. (PDF) [file pone.0182130.s007.pdf]

A

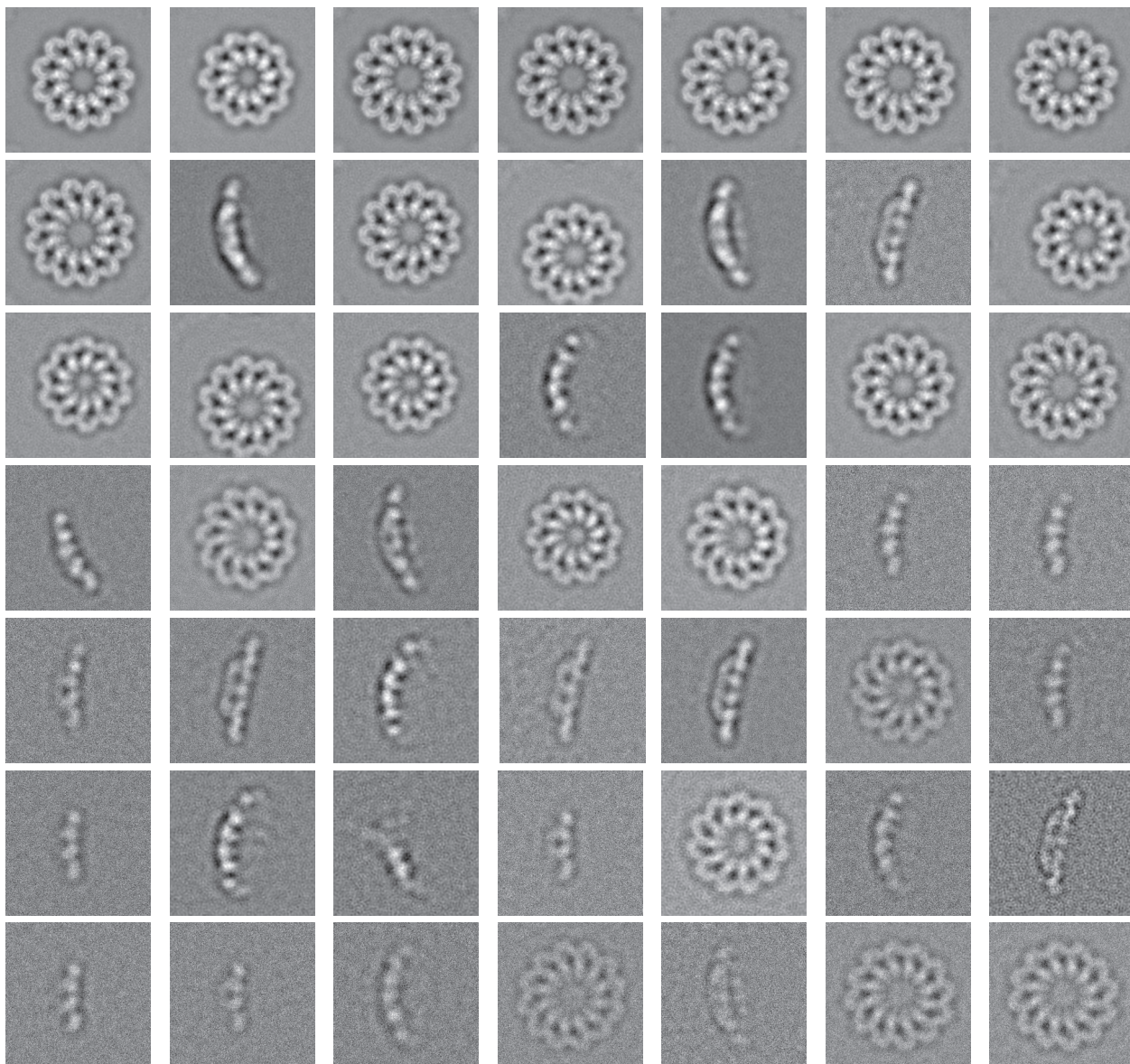

B

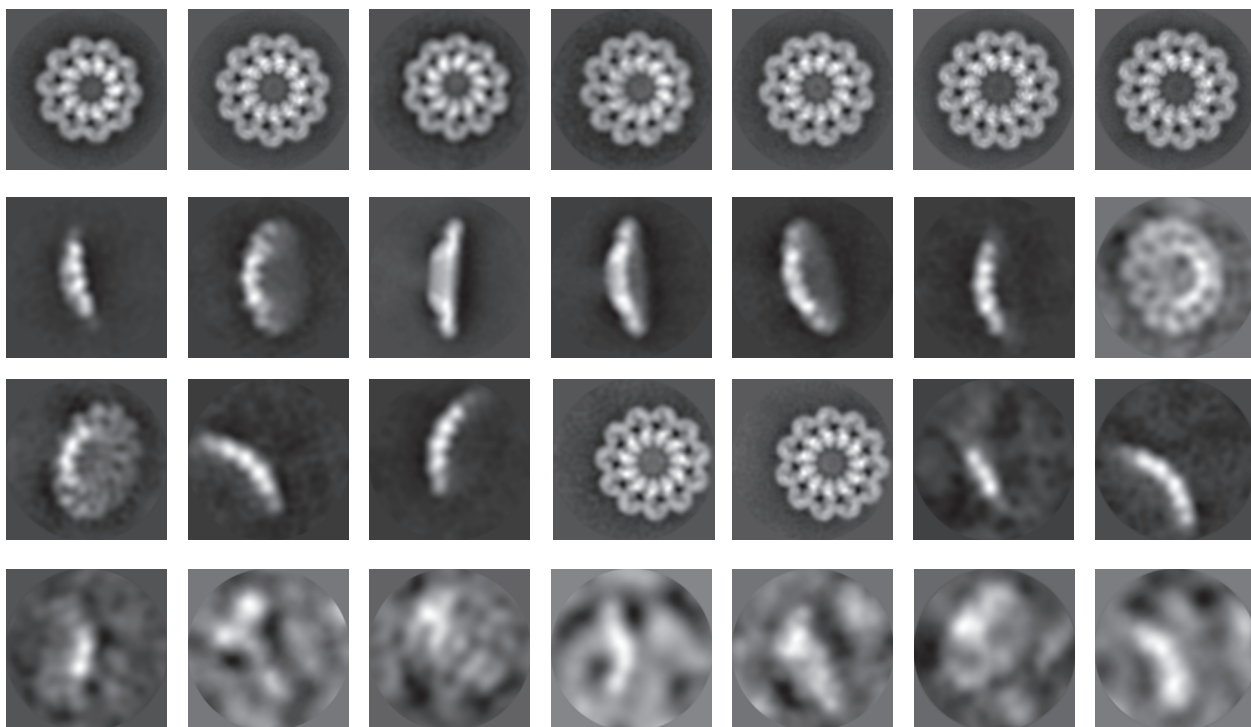

Supplement: S8 Fig — 17,103 particles of inflammasome were classified into 300 reference-free classes. Only classes whose particle numbers were greater than 9 were shown. (A) Unsupervised clustering using GTM in ROME. Among 300 classes, 49 classes showed the various views of inflammasome complexes with different symmetry. (B) Unsupervised classification using the MAP2D procedure in RELION. Only 20 classes exhibited views of inflammasome complexes with different symmetry. The MAP2D in RELION generated significantly fewer effective classes than did the GTM in ROME, indicating that GTM is more efficient for distinguishing structural differences. (PDF) [file pone.0182130.s008.pdf]

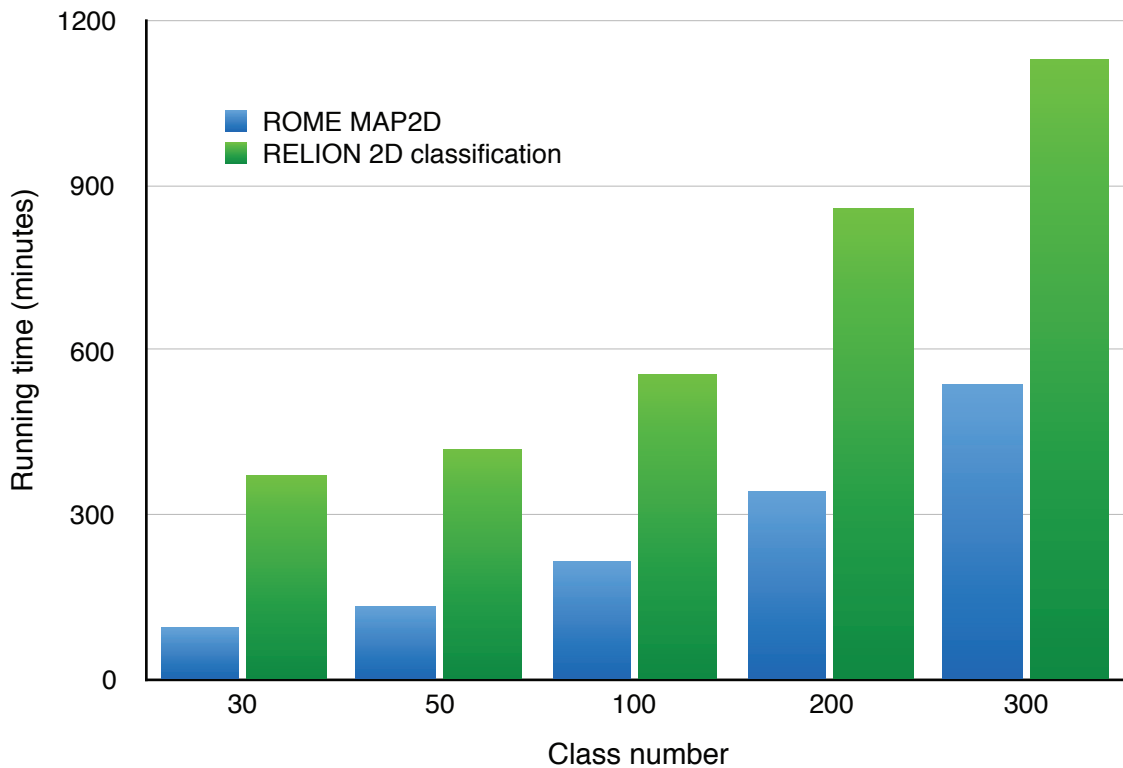

Supplement: S9 Fig — A 96,488-particle dataset of the RP-CP subcomplex particles with a box size of 180×180 pixels was used to test the performance of MAP2D in RELION 1.3 and MAP2D in ROME 1.0. When increasing the class number from 30 to 300, the running time of MAP2D classification in ROME followed a polynomial behavior from 92 to 537 minutes (blue histogram). (PDF) [file pone.0182130.s009.pdf]

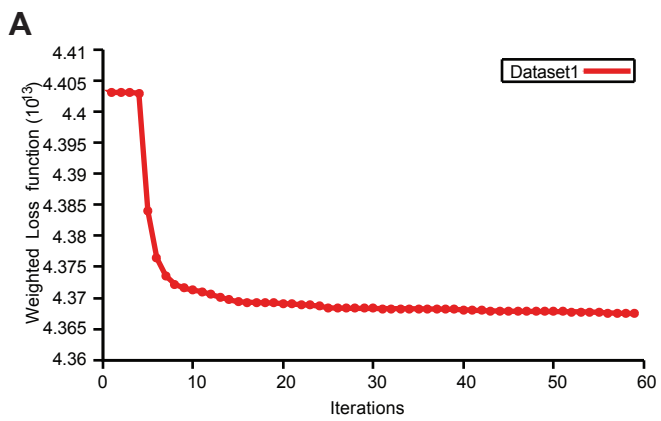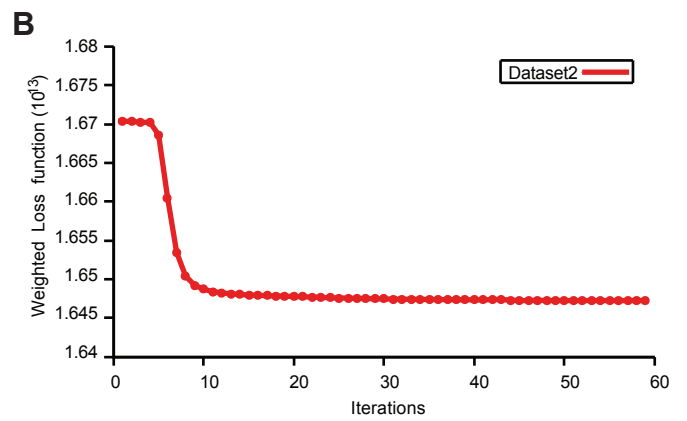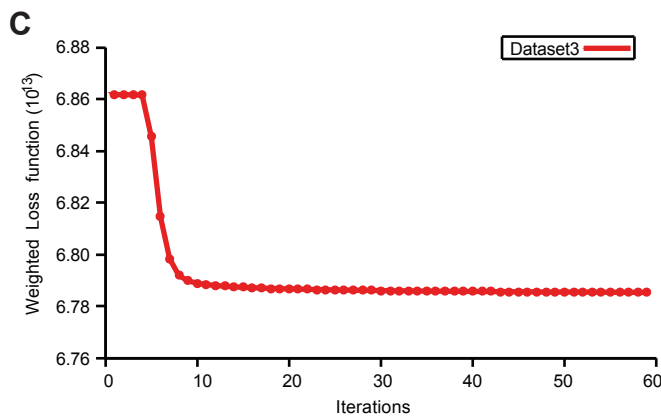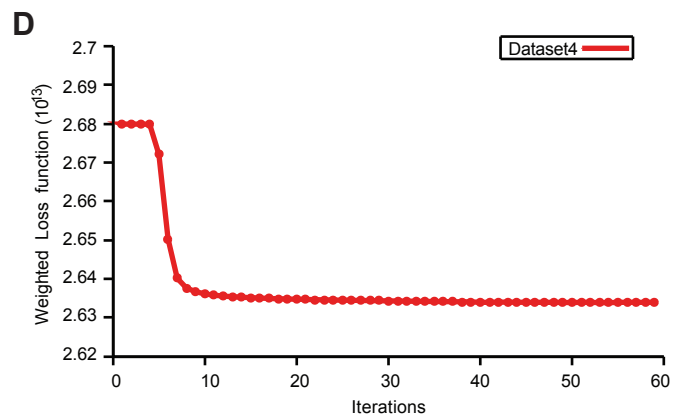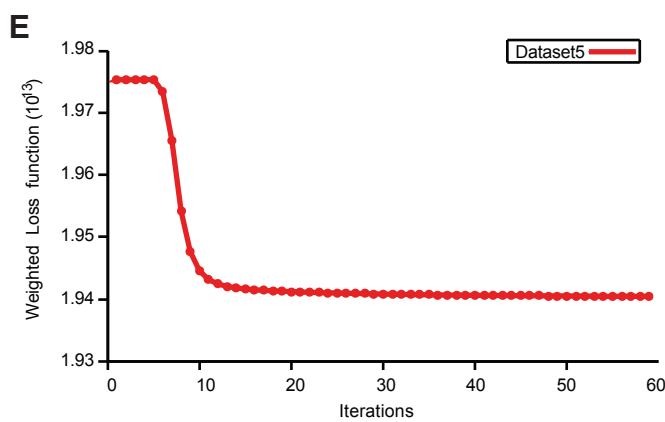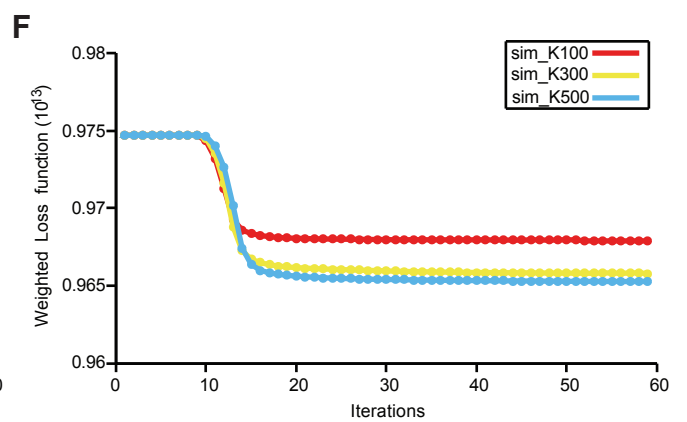

Supplement: S10 Fig — (A-D) The weighted loss function at each iteration for datasets 1 (panel A), 2 (panel B), 3 (panel C) and 4 (panel D). The four datasets were classified into 300 classes by GTM. (E) The dataset 5 was classified into 1,000 classes. The results of the weighted loss function at each iteration are shown. (F) The simulated data were classified into 100, 300 and 500 classes. The results of the weighted loss function at each iteration are shown. (PDF) [file pone.0182130.s010.pdf]
