# Supplementary material for: Massively parallel unsupervised single-particle cryo-EM data clustering via statistical manifold learning
Source: PLoS One. 2017 Aug 7;12(8):e0182130. doi: 10.1371/journal.pone.0182130 (PMC5546606; doi:10.1371/journal.pone.0182130)
Supplement: S1 Table — All tests were executed on the same computing cluster (32 nodes) consisting of 512 Intel Xeon E5 CPU cores. Based on the convergence criteria using the weighted loss function, all datasets converged within 25 iterations (see S10 Fig). RELION in the table refers to the unsupervised MAP2D-based classification in RELION 1.3. ROME_MAP in the table refers to the unsupervised 2D classification based on MAP2D methods implemented in ROME. ROME_SML in the table refers to the unsupervised 2D classification based on the GTM method implemented in ROME. In all cases, the E-M algorithm was run for 30 iterations. (PDF) [file pone.0182130.s011.pdf]

S1 Table

Software	class number	time(minutes)	data	images number	pixels
RELION	300	307	Dataset 1	16306	250*250
ROME_MAP	30	12	Dataset 1	16306	250*250
ROME_MAP(K=30)+gtm	300	20	Dataset 1	16306	250*250
RELION	300	880	Dataset 2	35407	160*160
ROME_MAP	30	56	Dataset 2	35407	160*160
ROME_MAP(K=30)+gtm	300	63	Dataset 2	35407	160*160
RELION	30	372	Dataset 3	96488	180*180
RELION	50	421	Dataset 3	96488	180*180
RELION	100	555	Dataset 3	96488	180*180
RELION	200	859	Dataset 3	96488	180*180
RELION	300	1132	Dataset 3	96488	180*180
ROME_MAP	30	92	Dataset 3	96488	180*180
ROME_MAP	50	130	Dataset 3	96488	180*180
ROME_MAP	100	216	Dataset 3	96488	180*180
ROME_MAP	200	340	Dataset 3	96488	180*180
ROME_MAP	300	537	Dataset 3	96488	180*180
ROME_SML (MAP_K=30)	100	14	Dataset 3	96488	180*180
ROME_SML (MAP_K=30)	300	20	Dataset 3	96488	180*180
ROME_SML (MAP_K=30)	500	28	Dataset 3	96488	180*180
ROME_SML (MAP_K=30)	700	75	Dataset 3	96488	180*180
ROME_SML (MAP_K=30)	800	94	Dataset 3	96488	180*180
ROME_SML (MAP_K=30)	900	167	Dataset 3	96488	180*180
ROME_SML (MAP_K=30)	1000	214	Dataset 3	96488	180*180
RELION	300	2379	Dataset 4	57001	160*160
ROME_MAP	30	134	Dataset 4	57001	160*160
ROME_MAP(K=30)+SML	300	143	Dataset 4	57001	160*160
ROME_MAP(K=100)+SML	1000	228	Dataset 5	117471	128*128
